# The Epigenetics of Neuropathic Pain: A Systematic Update

**DOI:** 10.3390/ijms242417143

**Published:** 2023-12-05

**Authors:** Gábor Pethő, Boglárka Kántás, Ádám Horváth, Erika Pintér

**Affiliations:** 1 Department of Pharmacology and Pharmacotherapy, Medical School, University of Pécs, Szigeti Str. 12., H-7624 Pécs, Hungary; boglarka.kantas@aok.pte.hu (B.K.); erika.pinter@aok.pte.hu (E.P.); 2 Department of Pharmacology, Faculty of Pharmacy, University of Pécs, Rókus Str. 2., H-7624 Pécs, Hungary; horvath.adam2@pte.hu; 3 Department of Obstetrics and Gynecology, University of Pécs, Édesanyák Str. 17., H-7624 Pécs, Hungary

**Keywords:** animal models, DNA methylation, epigenetics, histone acetylation, histone methylation, neuropathic pain, nociception

## Abstract

Epigenetics deals with alterations to the gene expression that occur without change in the nucleotide sequence in the DNA. Various covalent modifications of the DNA and/or the surrounding histone proteins have been revealed, including DNA methylation, histone acetylation, and methylation, which can either stimulate or inhibit protein expression at the transcriptional level. In the past decade, an exponentially increasing amount of data has been published on the association between epigenetic changes and the pathomechanism of pain, including its most challenging form, neuropathic pain. Epigenetic regulation of the chromatin by writer, reader, and eraser proteins has been revealed for diverse protein targets involved in the pathomechanism of neuropathic pain. They include receptors, ion channels, transporters, enzymes, cytokines, chemokines, growth factors, inflammasome proteins, etc. Most work has been invested in clarifying the epigenetic downregulation of mu opioid receptors and various K^+^ channels, two types of structures mediating neuronal inhibition. Conversely, epigenetic upregulation has been revealed for glutamate receptors, growth factors, and lymphokines involved in neuronal excitation. All these data cannot only help better understand the development of neuropathic pain but outline epigenetic writers, readers, and erasers whose pharmacological inhibition may represent a novel option in the treatment of pain.

## 1. Basic Concepts of Epigenetics

It is an old recognition that different cell types of the body that possess the same nucleotide sequence in the deoxyribonucleic acid (DNA) have distinct structures and functions, and these features are primarily determined by differences in the expression profile of the genes, leading to changes in the cellular amount and function of proteins. Epigenetics deals with modifications to the gene expression that occur without alteration of the nucleotide sequence in response to external (diet, lifestyle, eventual drug exposure, etc.) or internal factors (e.g., sex, race, development, aging, pathological states). Epigenetic processes can be initiated by altering the chromatin substance composed of DNA and histone proteins. Chromatin is built up from repeating structural units called nucleosomes. In each nucleosome, about 140 nucleotide-pair-long DNA is wrapped around an octamer complex of histone proteins, including 2–2 copies of H2A, H2B, H3, and H4. The H1 and H2 proteins are bound to the DNA as it enters the nucleosomes and keep in place the DNA that has wrapped around the nucleosome core, and they also bind to the linker DNA connecting the adjacent nucleosomes. Chromatin can exist in two forms. Heterochromatin is a condensed configuration with short free segments of DNA (forming a beads-on-a-string structure) repressing transcription. Euchromatin is a more relaxed configuration in which longer free DNA segments allow for the binding of transcription factors, thus promoting transcription.

Epigenetic processes lead to covalent modifications of the DNA in the form of methylation or alteration of the histone proteins by methylation, acetylation, phosphorylation, etc., which result in an increase or decrease in gene expression, typically at the transcriptional level [1]. Several enzymes responsible for attaching and removing these epigenetic molecular marks (so-called writers and erasers, respectively) and protein factors recognizing them (so-called readers) have been identified and characterized. However, much less is known about how the various extracellular and intracellular signals can facilitate the activity of the writers. Similarly, the intracellular events following the interaction between the readers and their downstream factors have only been partially elucidated.

## 2. The Role of DNA Methylation/Demethylation in Neuropathic Pain

### 2.1. General Principles

DNA methyltransferase (DNMT) enzymes attach a methyl group to carbon 5 of a cytosine base adjacent to a guanine residue, a so-called CpG site, which is typically concentrated at the promoter region of genes that form CpG islands [2]. There are several forms of DNMTs; DNMT1, as a maintenance enzyme, functions during cell division to copy the methylation pattern of the old DNA strand onto the complementary new one, whereas DNMT3a and DNMT3b mediate de novo methylation in response to the above-mentioned external and internal factors. Methylation of CpG islands typically causes gene repression, partly by preventing the binding of transcription factors and partly by functioning as docking sites for 70 amino acid-long methyl-CpG-binding domain (MBD) proteins, inhibiting transcription. These epigenetic readers include methyl-CpG-binding protein 2 (MeCP2) and a series of MBD 1–6 proteins, and they work by recruiting other transcriptional repressors such as histone deacetylase or histone methyltransferase enzymes (see later). DNMT2 is a homolog that methylates cytosine bases in the transfer RNA.

DNA demethylation can be passive or active. The passive way refers to the absence of methylation of newly synthesized DNA strands by DNMT1 during repeated DNA replication cycles. Active demethylation occurs without DNA replication by 10 to 11 translocation (TET) methylcytosine dioxygenase enzymes, which are so-called editors that are able to modify epigenetic marks. TET enzymes convert 5-methylcytosine (5mC) to 5-hydroxymethylcytosine (5hmC) in the CpG dinucleotide. Furthermore, TET1 itself, as well as the formed 5-hydroxymethylcytosine, both prevent the binding of DNMT to CpG sites. As these activities contribute to DNA demethylation, TET enzymes can also be regarded as epigenetic erasers [3].

### 2.2. Expression of Elements of the DNA Methylation Machinery in Primary Sensory Neurons

Proteins involved in DNA methylation/demethylation have been localized to the dorsal root ganglia (DRG) that contains the cell body of primary sensory neurons, including nociceptive ones, and in the spinal dorsal horn, comprising the central terminals of (nociceptive) primary afferent neurons and the bodies of other cell types. Concerning the writer proteins, all three forms of DNMTs (DNMT1, DNMT3a, and DNMT3b) are expressed in the DRGs under normal conditions [4,5,6,7]. A predominantly neuronal expression in both peptidergic and non-peptidergic primary sensory neurons was detected. DNMT3a and DNMT3b were also revealed in the dorsal horn [8]. Considering the reader proteins, MeCP2 expression in the lumbar spinal cord of rats was revealed—mainly in neurons, much less in glial cells [4,9]. Similarly, MBD1 expression was detected in both peptidergic and non-peptidergic sensory neurons, but not in glial cells of the mouse DRG [10]. Regarding the eraser proteins, TET proteins were also detected in the DRG of mice: TET1 and TET3 only in neurons, whereas TET2 was also in non-neuronal cells; TET3 was predominantly located in presumably nociceptive small- and medium-sized neurons [11]. Accordingly, a 5hmC signal was detected in nearly all DRG neurons of mice. TET1 expression was also observed in the dorsal horn neurons of rats [12].

### 2.3. Global Changes in DNA Methylation under Neuropathic Conditions

An early study on rats with chronic constriction injury (CCI) of the sciatic nerve revealed an increased global DNA methylation and upregulation of MeCP2 in the lumbar spinal cord on day 14 after nerve injury [9]. These changes were inhibited by intrathecally applied 5-azacytidine, a nonselective inhibitor of DNMT enzymes. The latter agent also reduced CCI-evoked mechanical allodynia and heat hyperalgesia, suggesting that CCI may lead to epigenetic silencing of some putative antinociceptive genes via DNA methylation. In a genome-wide analysis of the spinal cords of rats with CCI, 638 hypermethylated and 567 hypomethylated sites within promoter regions were revealed [13].

One day after spinal nerve ligation (SNL), in DRGs of rats, plastic changes of the DNA methylation pattern were revealed with predominant (80%) hypermethylation of CpG sites observed, not only at the promoter regions of genes, but also in exons and introns as well as outside of genes [14]. There was no clear correlation between the direction of change of the methylation status and the level of gene expression. In the same model, the genome-wide analysis revealed both DNA hypermethylation and hypomethylation in the affected DRGs 3 days after injury [15]. In contrast, three weeks after injury, DNA hypomethylation prevailed predominantly outside CpG islands and in introns, intergenic regions, and repeats. Interestingly, DNA hypermethylation was more typical in the spinal cord and prefrontal cortex. The nerve injury-induced methylation reprogramming correlated with increased gene expression in the DRGs. These data suggest that DNA hypomethylation in the DRG prevails in this model of neuropathic pain.

In the spared nerve injury (SNI) model of rats, involving ligation of the tibial and peroneal nerves but sparing the sural nerve, messenger ribonucleic acid (mRNA) expressions of DNMTs and MeCP2 were downregulated in the dorsal horn, the latter also in the ipsilateral, injured DRG [4]. In accordance, the 5hmC signal, indicating DNA demethylation, was increased at the ipsilateral DRGs of mice with SNI, and TET3 expression was increased, as opposed to TET1 or TET2 expression [11]. All these alterations point to nerve injury-evoked DNA hypomethylation in this model. The genome-wide analysis in mice with SNI revealed in the DRG a redistribution of MeCP2 binding to transcriptionally relevant regions [16].

The prefrontal cortex is of crucial importance for chronic pain states as an integrator area. In early studies in this field employing the SNI model in mice or rats six or nine months after nerve injury, a decreased global DNA methylation level was detected in the prefrontal cortex and amygdala, but not in other brain areas [17,18]. Accordingly, DNA hypomethylation in this structure correlated with mechanical allodynia and cold hyperalgesia. Chronic post-treatment of SNI mice with the methyl donor S-adenosylmethionine diminished nerve injury-induced mechanical hypersensitivity, indirectly supporting the role of DNA methylation [19]. In a long-term follow-up study on SNI mice, the global DNA methylation pattern in the prefrontal cortex was assessed 1 day, 2 weeks, 6 months, and 1 year after nerve injury [20]. Over this long period, time point-specific differential methylation of individual genes at the promoter regions was revealed, affecting numerous pain-related genes. In rats with SNI, TET1 expression was increased in the prefrontal cortex, and DNMT1 expression was increased in the hippocampus [21,22]. In the partial sciatic nerve ligation (PSNL) model of mice, the reduced global DNA methylation in the prefrontal cortex and in the periaqueductal grey matter, which has been confirmed and correlated with mechanical allodynia and cold allodynia [23]. Using differentially methylated regions analysis, 2451 hypermethylated and 1991 hypomethylated gene promoters were identified. In addition, expressions of DNMT1, DNMT3a, and DNMT3b were decreased as well. Acupuncture could restore both the DNA hypomethylation in the prefrontal cortex and the nociceptive hyperresponsiveness. In a mouse model of neuropathic pain based on unilateral transection of the tibial nerve, global DNA methylation was increased bilaterally in the primary somatosensory cortex [24].

Paclitaxel-induced neuropathy caused no significant alterations in DNA methylation levels in the DRG, but, in mice with streptozotocin-induced diabetes, 376 genes were hypermethylated and 336 were hypomethylated at CpG sites of promoter regions in the DRGs [15,25]. In a recent human study, the genome-wide analysis revealed differentially methylated regions in the DNA obtained from blood cells [26]. The methylation pattern of patients with neuropathic pain was different, not only compared to healthy controls, but also to patients suffering from nociceptive pain.

### 2.4. Proteins Regulated by DNA Methylation in Neuropathic Conditions

#### 2.4.1. Opioid Receptors

G_i_ protein-coupled mu, delta, and kappa opioid (MOP, DOP, and KOP, respectively) receptors mediate the analgesic effects of both therapeutically applied opioids and endogenous opioid peptides. In particular, the MOP receptor is subject to epigenetic regulation by DNA methylation. As shown in mice with CCI, morphine’s peripheral and spinal antinociceptive effects were decreased due to the downregulation of the MOP receptor in the DRG and the spinal cord [27,28]. Increased methylation at the proximal promoter of the MOP receptor gene was revealed in DRG neurons, along with MeCP2 upregulation. DNMT inhibition by 5-aza-deoxycytidine or knockdown of MeCP2 prevented MOP receptor downregulation in DRG and the spinal cord and restored the antinociceptive potency of morphine reduced by nerve injury. Furthermore, the octamer transcription factor 1 (OCT1) overexpression in DRG neurons was also involved in the CCI-evoked upregulation of DNMT3a and downregulation of MOP receptors [29]. Similar results were obtained regarding the spinal cord: CCI-induced heat hyperalgesia was associated with increased DNMT3a expression, the enhanced methylation level of the proximal promoter of the MOP receptor gene, and decreased MOP receptor expression [30]. All these alterations were reduced by intrathecally applied RG 108, a nonselective and irreversible DNMT inhibitor.

Consonant results were obtained in the SNL model of rats and mice. DNMT3a upregulation led to increased DNA methylation at the promoter region of the MOP receptor gene and downregulation of MOP (and KOP) receptors in the injured DRG, along with the development of tolerance to the antinociceptive effect of morphine [5,10]. In addition, a role for the reader protein MBD1 was also established, as it was upregulated in the injured DRG. DNMT3a was recruited by MBD1 to the promoter of the respective genes and involved in the transcriptional repression of the MOP receptor. In either MBD1 knockout or MBD1 knockdown (by short hairpin RNA (shRNA)) mice, the SNL-evoked mechanical allodynia, heat hyperalgesia, and cold allodynia were diminished. The SNL-induced promoter hypermethylation and downregulation of the MOP receptor gene were counteracted by microinjection of herpes simplex virus, expressing TET1 mRNA into the injured DRG [31]. This overexpression of TET1 restored the diminished analgesic efficacy of morphine and counteracted morphine tolerance induced by nerve injury.

All the above data provide evidence that hypermethylation of the MOP receptor gene promoter leads to transcriptional repression of MOP receptors with two consequences: induction of hyperalgesia, likely by reducing the antinociceptive effects of endogenous opioids, along with a diminishment of the antinociceptive effect of the applied opioid.

#### 2.4.2. K^+^ Channels (K_v_1.2, K_2p_1.1)

K^+^ channel opening typically causes an outward current, leading to membrane hyperpolarization and consequent neuronal inhibition. Conversely, inhibition or downregulation of K^+^ channels induces neuronal hyperexcitability via different mechanisms, including reduction of the total K^+^ current, increasing the resting membrane potential, reducing the current threshold for action potential, and increasing the number of evoked action potentials. Extensive evidence exists that decreased expression of various K^+^ channels contributes to neuropathic pain. For two types of them, the voltage-gated K_v_1.2 channels and the two-pore-domain K^+^ channels (K_2P_), epigenetic regulation by DNA methylation in neuropathy has been revealed. In rats with SNL and/or CCI, an increased expression of DNMT3a but not DNMT3b in the injured DRG was observed [6,29]. Evidence has been provided that upregulation of OCT1 in the corresponding ipsilateral DRG but not in the spinal cord was involved in this response. OCT1 was predominantly localized in both large and small DRG neurons, including peptidergic and non-peptidergic small ones. In the DRG, DNMT3a directly inhibits the expression of the K_v_1.2 channels by methylation at the promoter region of the K_v_1.2 gene. These data suggest that nerve injury upregulates DNMT3a in DRG neurons involving OCT1 action, which leads to enhanced methylation of the promoter of the K_v_1.2 gene, resulting in K_v_1.2 downregulation. A role for the reader protein MBD1 was also revealed in mice with SNL [10]. It was shown that DNMT3a was recruited by the upregulated MBD1 to the promoter of the K_v_1.2 channel gene and was involved in the transcriptional repression of this channel. The SNL-induced promoter hypermethylation and downregulation of the K_v_1.2 gene were rescued by artificial TET1 overexpression in the injured DRG [31].

Similar results were obtained upon implantation of prostate cancer cells into the rat tibia, which led to mechanical allodynia, heat hyperalgesia, and cold allodynia, along with increased DNMT3a (but not DNMT3b) expression and a decreased K_v_1.2 channel expression in the ipsilateral dorsal horn (but not DRG) [8]. Pharmacological inhibition (by decitabine, a nonselective DNMT inhibitor) or genetic knockdown (by shRNA) of DNMT3a prevented tumor cell-induced K_v_1.2 channel downregulation and reversed the behavioral responses.

In a complex study employing SNL, CCI, and axotomy models, evidence for involvement of DNMT1, the primary maintenance DNMT, in neuropathic pain has been provided [7]. DNMT1 was upregulated in the DRG in response to nerve injury through the transcription factor cyclic adenosine monophosphate (AMP) response element-binding (CREB) protein, which was shown to directly bind to the promoter of the DNMT1 gene, leading to transcriptional activation. DNMT1 was shown to reduce K_v_1.2 channel expression (but not MOP or KOP receptor levels) in the injured DRG by methylating three sites of the K_v_1.2 gene. These data suggest that DNMT1 may be induced under neuropathic conditions and mediate de novo methylation of the K_v_1.2 gene.

Systemic paclitaxel administration in mice induced downregulation in the DRGs of K_2p_1.1, a member of the K_2P_ family involved in the leak currents of neurons [32]. Evidence has been provided that K_2p_1.1 downregulation leads to increased neuronal excitability in DRG neurons and pain hypersensitivity. In DRG, paclitaxel treatment evoked an increased expression of DNMT3a but not DNMT3b or DNMT1. Finally, paclitaxel increased DNA methylation at the promoter region of the K_2p_1.1 gene. These data suggest that paclitaxel upregulates DNMT3a in DRG neurons, leading to enhanced methylation of the K_2p_1.1 promoter, resulting in K_2p_1.1 downregulation and neuronal excitation.

#### 2.4.3. Brain-Derived Neurotrophic Factor

Brain-derived neurotrophic factor (BDNF) is released from central terminals of the nociceptive primary sensory neurons and contributes to spinal sensitization of the pain pathway. In rats with SNL, increased expression of TET1, peaking on day 7, was observed in the ipsilateral dorsal horn neurons [12]. As expected, SNL increased the global level of 5hmC in the ipsilateral dorsal horn, suggesting that SNL-increased spinal TET1 expression promotes DNA demethylation. SNL increased the conversion of 5mC to 5hmC at the CpG sites of the promoter of the BDNF gene. SNL increased the levels of BDNF in the dorsal horn, and this alteration was reduced by spinal TET1 knockdown. SNL-enhanced DNMT1, DNMT3a, and DNMT3b binding to the promoter of the BDNF gene were reduced on day 7 compared to day 3. TET1 knockdown increased DNMT1, DNMT3a, and DNMT3b binding to the promoter of the BDNF gene on day 7. These data indicate that, initially, methylation of the BDNF gene promoter is enhanced by DNMT enzymes, but later it is overcompensated by an increased spinal TET1 expression, partly by direct demethylation, partly by reducing DNMT binding. MeCP2 was shown to be upregulated in the SNI model of mice, and it was revealed that functional MeCP2 is needed for regular BDNF expression in DRG [33].

#### 2.4.4. Other Proteins

The following epigenetic mechanism has been revealed regarding glutamic acid decarboxylase 67 (GAD67), an enzyme converting the main neuroexcitatory mediator glutamate to the main inhibitory neurotransmitter gamma-aminobutyric acid (GABA). In rats with CCI, expression of DNMT3a, DNMT3b, and MeCP2 increased, whereas MBD2 expression decreased in the lumbar spinal cord [34]. These changes were associated with enhanced DNA methylation at the promoter of the GAD67 gene and downregulation of GAD67.

In rats with SNL, an upregulation of TET1 in the dorsal horn, but not the DRG, was found, which led to hypomethylation of the metabotropic glutamate receptor (mGlu) type 5 (mGlu5) gene promoter, causing increased expression of this receptor in the dorsal horn, with consequent mechanical allodynia and heat hyperalgesia [35]. It is worth mentioning that, in the same model, TET1 overexpression in the injured DRG (but not the spinal cord) by viral delivery of TET1 mRNA alleviated heat hyperalgesia and mechanical allodynia in rats [31].

The methylation levels of the genes of the metabotropic glutamate receptor type 4 (mGlu4), serotonin 5-HT_4_ receptor, β_2_ adrenergic receptor, and K_v_5.1 channel were increased in both the CCI and SNL models of neuropathic pain in the rat [13]. Accordingly, the mRNAs of these receptors/channels were downregulated in the spinal cord. Intraperitoneally applied 5-azacytidine, a DNMT inhibitor, reversed the above-mentioned DNA and mRNA alterations and diminished neuropathic mechanical allodynia and heat hyperalgesia.

Regarding diabetic neuropathy, streptozotocin-induced diabetes attenuated the expression of DNMT3b, but not 3a, in the DRG, leading to demethylation at the promoter of the P2X3 purinoceptor gene [36]. This resulted in increased binding of p65, a subunit of the transcription factor nuclear factor kappa B (NF-κB), to the demethylated promoter region, which increased P2X3 channel expression in DRG neurons, contributing to mechanical allodynia. The nucleotide oligomerization domain (NOD)-like receptor protein 3 (NLRP3), a core inflammasome component, was upregulated in the DRGs of mice with streptozotocin-induced diabetes, and this latter alteration contributed to neuropathic mechanical allodynia [37]. The likely mechanism is the upregulation of TET2, leading to an increase in thioredoxin-interacting protein (TXNIP) expression in neurons, which can activate the NLRP3 inflammasome.

In various tumors, including oral squamous cell carcinoma, endothelin levels are increased. Endothelin acting at its type A receptors promotes nociception, whereas activation of type B receptors results in antinociception. In a human oral squamous cell carcinoma specimen, an elevated methylation level was detected at the promoter of the endothelin type B (ET_B_) receptor gene, along with reduced expression of ET_B_ [38]. Restoring ET_B_ receptor expression by a viral vector of the ET_B_ gene in the tumor cells led to decreased endothelin secretion but increased beta-endorphin secretion. In a murine oral squamous cell carcinoma model, overexpression of the ET_B_ receptor gene attenuated mechanical allodynia without affecting tumor size.

In oxaliplatin-treated rats, the transcription factor SRY-related HMG-box 10 (SOX10) was upregulated due to hypomethylation of the SOX10 gene promoter by upregulated TET1 [39]. Increased expression and binding of SOX10 to the promoter of the gene of the transcription factor homeobox A6 (HOXA6) protein led to HOXA6 upregulation in spinal dorsal horn neurons, which contributed to oxaliplatin-induced mechanical allodynia. Oxaliplatin-induced mechanical allodynia was shown to involve increased expression of the transcriptional regulator zinc-finger E-box-binding homeobox 1 (ZEB1) and the DNMT3b in the dorsal horn neurons of the rat [40]. Their enhanced interaction increased the methylation level at the promoter of the gene of the tyrosin kinase-linked receptor discoidin domain receptor type 1 (DDR1). This led to a decrease in DDR1 expression and mechanical allodynia.

In the intervertebral discs of mice, expression of secreted protein acidic and rich in cysteine (SPARC, also known as basement-membrane protein 40 or osteonectin) was shown to decrease with age, and this decline was associated with nociceptive responses resembling both axial and radicular low back pain of humans [41]. This age-dependent downregulation of SPARC was coupled with increased methylation of six CpG sites at the promoter of the SPARC gene. In addition, 5-azacytidine, a DNMT inhibitor, increased SPARC expression. In patients with chronic neuropathic pain, a correlation between transient receptor potential (TRP) ankyrin 1 (TRPA1) gene hypermethylation or TRPA1 mRNA downregulation in blood cells and pain score was established [42].

Epigenetically relevant details of the neuropathy models in which DNA methylation was investigated are shown in Table 1.

## 3. Histone Protein Modification by Acetylation/Deacetylation under Neuropathic Conditions

### 3.1. General Principles

The most extensively studied epigenetic modification to the histone proteins is acetylation occurring at lysine residues of the amino-terminal tail of histone molecules protruding from the nucleosomes [43,44]. This posttranslational alteration loosens the compact structure of the DNA, leading to the formation of euchromatin, thereby causing transcriptional activation. In addition, acetylated histone tails can serve as markers that attract various transcription factors, which is another method of transcriptional regulation. Histone acetyltransferase (HAT) enzymes confer an acetyl group onto the tail of the histone proteins. They can be classified as A-type HATs having numerous subtypes, including p300/CREB binding protein (CBP), general control non-derepressible 5 related acetyltransferases (GNAT), MYST, and other families, each comprising several isoforms and exerting histone acetylation in the nuclear chromatin. B-type HATs are located in the cytoplasm, and they acetylate de novo synthesized histone proteins, which are then transported to the nucleus. HAT isoenzymes exhibit different degrees of selectivity regarding target histones, with p300/CBP having the broadest substrate spectrum, as it can acetylate all types of histone subunits in the nucleosome. HAT inhibitors also possess different subtype selectivity, and they include, among others, curcumin, anacardic acid, and garcinol. Reader elements of this epigenetic machinery have also been identified. Bromodomain refers to an approximately 110 amino acid-long part of a protein, which can recognize acetylated lysine residues in histone or non-histone proteins. Bromodomain-containing proteins (such as bromodomain and extraterminal [BET] domain [BRD] proteins, including BRD2, BRD3, BRD4, and BRDT) are typical epigenetic readers possessing diverse functions, including HAT activity or direct transcriptional regulation.

Deacetylation of the histone tail induces compaction of the DNA, i.e., heterochromatin formation resulting in transcriptional repression. Histone deacetylase (HDAC) enzymes, being typical epigenetic erasers, remove the acetyl group from the histone tail, leading to heterochromatin formation. They are classified into four groups: Class I (including HDAC1, HDAC2, HDAC3, and HDAC8), class IIa (including HDAC4, HDAC5, HDAC7, and HDAC9), class IIb (including HDAC6 and HDAC10), and class IV (including HDAC11) enzymes, which hydrolyze the amide bond on the acetylated lysine residues using zinc ions as a cofactor. In contrast, class III HDAC enzymes, including silent information regulators (SIRT), so-called sirtuins (SIRT1–7), transfer the acetyl group from the acetylated lysine to nicotinamide adenine dinucleotide (NAD+). Most HDACs can be found either in the nucleus or in the cytoplasm. In contrast, class IIa HDACs shuttle between the nucleus and cytoplasm, which is regulated by phosphorylation at lysine residues. The phosphorylated form is extruded from the nucleus and, thus, remains in the cytoplasm. However, upon cytoplasmic dephosphorylation, it can be transported back to the nucleus. HDAC inhibitors, including sodium butyrate, trichostatin A, valproic acid, suberoylanilide hydroxamic acid (SAHA), etc., are also available similarly to SIRT inhibitors (nicotinamide, sirtinol, splitomicin). Interestingly, activators of SIRT enzymes such as resveratrol have also been identified. It must be emphasized that some HATs and HDACs can acetylate/deacetylate non-histone proteins, including NF-κB, making interpretation of the results obtained with HAT or HDAC inhibitors biased concerning epigenetic relevance.

### 3.2. Expression of Critical Elements of the Histone Acetylation Machinery in Nociceptive Primary Sensory Neurons

Expressions of HDAC1, HDAC2, HDAC4, and HDAC5 were revealed in rat DRG, with HDAC2, HDAC4, and HDAC5 being predominantly localized in neurons [45]. HDAC2, HDAC4, and HDAC5 were upregulated by SNL injury. HDAC2 expression has been revealed in dorsal horn neurons and, to a lesser extent, in astrocytes, but not in the microglia of rats [46]. Seven days following SNI, an increase in HDAC2 expression could be detected in spinal astrocytes but not neurons. In a rat model of bone cancer pain, HDAC1 was upregulated in neurons and astrocytes in the dorsal horn, while HDAC2 was upregulated in astrocytes [47]. In DRG, expression of both HDAC1 and HDAC2 was increased mainly in satellite glial cells. SIRT1 expression was revealed in spinal neurons but not glial cells [48].

### 3.3. Global Epigenetic Alterations Involving Histone Acetylation/Deacetylation in Animal Models of Neuropathic Pain

In the PSNL model of mice, the number of HDAC1-positive microglia in the ipsilateral dorsal horn was increased, and the acetylation of histone H3 at lysine (H3K) 9 residue (H3K9) in activated microglia was decreased [49]. These responses were reversed by treadmill running, along with a reduction of mechanical allodynia and heat hyperalgesia [49]. In rats with either PSNL or SNL (similarly to stavudine treatment), two different HDAC inhibitors (MS-275, MGCD0103) delivered intrathecally reduced nerve injury-induced mechanical and heat hyperalgesia, but only if HDAC inhibitor treatment preceded neuropathy induction [50]. The drugs increased global H3K9 acetylation in the spinal cord but not the DRG. In rats with SNL, increased HDAC1 expression and reduced H3 acetylation were measured in the dorsal horn [51]. Intrathecal treatment by the flavonoid baicalin reversed both of these changes, along with the diminishment of the tactile allodynia and heat hyperalgesia. In rats with CCI, sodium butyrate, an HDAC I and IIa inhibitor given orally for 14 days, diminished cold and mechanical allodynia, as well as heat and mechanical hyperalgesia [52]. In mice with SNL, mechanical allodynia was accompanied by cytoplasmic retention of HDAC4 in the ipsilateral dorsal horn neurons [53]. Nerve injury activated serum- and glucocorticoid-inducible kinase 1 (SGK1), which phosphorylates HDAC4 in the nucleus. Phosphorylated HDAC4 is exported from the nucleus to the cytoplasm, where it interacts with 14-3-3β, a phosphor-binding protein that acts as an anchoring element, which prevents its transport back to the nucleus. Cytoplasmic retention of HDAC4 prevents its ability to inhibit transcription in the nucleus, leading to possible upregulation of pronociceptive proteins.

In the SNI model of mice, HDAC1 and HDAC2 were upregulated in the spinal cord, but the expression level of HDAC3 remained unaltered [46,54,55,56]. The selective HDAC1 inhibitor LG325, applied intrathecally, reversed the upregulation of HDAC1 in the spinal cord and reduced mechanical allodynia [54]. Another HDAC1-selective inhibitor, zingiberene, given orally evoked the same effects and reduced heat hyperalgesia [56]. Combined inhibition of HDAC enzymes and BET proteins by intranasally applied inhibitors resulted in additive or even potentiating interactions in the SNI model, with a decrease in HDAC1, the reader protein BRD4 expression, microglia activation, spinal neuroinflammation, and mechanical allodynia, as well as heat hyperalgesia [57,58]. In the same model, nerve injury activated the mitogen-activated protein kinase C-Jun N-terminal kinase (JNK) 1 in the spinal cord [55]. JNK1 activation led to upregulation of HDAC1, which activates c-Jun. Direct evidence for the interaction between HDAC1 and c-Jun was also provided. However, in the SNI model of rats, a downregulation of HDAC1–3 was reported [4]. In mice with SNI, histone alterations (H3K4me1, H3K4me4, and H3K27ac) were examined in different brain areas related to pain [59]. In the periaqeductal grey matter, H3K4me1 levels were decreased. In the lateral hypothalamus H3K27ac levels were decreased. In the nucleus accumbens H3K27ac levels were also decreased. These chromatin alterations correlated with mechanical and thermal hypersensitivity caused by nerve injury.

As mentioned earlier, SIRT1 is a protein from the sirtuin family, a class III, NAD^+^-dependent HDAC. In rats or mice with CCI, heat hyperalgesia and mechanical allodynia were accompanied by SIRT1 downregulation in the spinal cord [60,61]. As expected, the H4 and H3 histone proteins exhibited an increased acetylation level. Intrathecally applied resveratrol, an activator of SIRT1, delayed the onset of hyperalgesia and allodynia, along with a reversal of histone hyperacetylation. Similar results have been obtained with SRT1720, a SIRT1 activator 1000 times more potent than resveratrol [62]. Inversely, a SIRT inhibitor prevented the antinociceptive actions of resveratrol. In rats with CCI, SRT1720 exerted antihyperalgesic/antiallodynic actions and decreased CCI-induced overexpression of the molecular target of rapamycin (mTOR), NF-κB, interleukin (IL) 6, tumor necrosis factor α (TNF-α), and inducible nitric oxide synthase (iNOS) [63]. CCI-induced upregulation of the protein factor erythroblast transformation specific (ETS) proto-oncogene I (ETSI) in small- and medium-sized DRG neurons was responsible for mechanical allodynia and heat hyperalgesia in mice [64]. ETSI was shown to induce HDAC1 upregulation through binding to its promoter, ultimately leading to an increased expression of phosphorylated extracellular signal-regulated kinase 1/2 (pERK1/2) and glial fibrillary acidic protein (GFAP). Downregulation of SIRT1 in the spinal cord was also revealed in the paclitaxel-induced neuropathy model in the rat, similar to bortezomib-evoked neuropathy [65]. It was associated with H4 hyperacetylation and NF-kB (p65) phosphorylation.

In models of orofacial pain/trigeminal neuralgia in mice or rats, a global decrease in H3K9 acetylation was observed in injured trigeminal ganglion neurons, along with an alteration of the expression level of a huge array of genes [66,67]. Preemptive HDAC inhibition by either SAHA, MS-275, or carbamazepine restored H3K9ac levels and prevented mechanical allodynia. It was shown that the transfer of phosphate groups from activated extracellular signal-regulated kinase (ERK) and leucine-reach repeat kinase 2 onto HDAC3 causes hyperactivity of the enzyme in this type of neuropathy [67]. In rats with CCI in the infraorbital nerve, SIRT1 downregulation was revealed in GABAergic neurons of the central nucleus of the amygdala [68]. In accordance, in this area, a hyperacetylation of the H3K9 at the promoter of the Ca^2+^/calmodulin-dependent protein kinase IIaα (CaMKIIaα) gene was observed, along with an upregulation of the enzyme.

### 3.4. Proteins Regulated by Histone Acetylation/Deacetylation in Neuropathic Conditions

#### 3.4.1. Neurotransmitters

Glutamate, the main excitatory neurotransmitter in the central nervous system, plays an important role in neurotransmission in the spinal dorsal horn, including central sensitization of the nociceptive transmission, thereby contributing to pain and hyperalgesia. GABA, the main inhibitory transmitter in the central nervous system, can counteract glutamate-mediated excitatory effects. Thus, the actual balance between the opposing glutamate-mediated and GABA-mediated synaptic activities determines the level of neural excitation in the spinal cord and brain.

In spinal neurons, but not glial cells, of type 2 diabetic rats, decreased expression and activity of SIRT1 was revealed [48]. This resulted in H3 hyperacetylation at the promoter region of the mGlu1/5 receptor genes, leading to increased expression of these receptors. In rats with SNL, expression of glutamic acid decarboxylase 65 (GAD65), an enzyme inactivating glutamate by converting it to GABA, was decreased in the nucleus raphe magnus of the brainstem, a critical site for the maintenance of hyperalgesia/allodynia [69]. Evidence has been provided that 3 weeks (but not 1 day) after injury, GAD65 downregulation was due to diminished H3 acetylation at the promoter region of the GAD65 gene. HDAC inhibitors restored the acetylation level and GABAergic neurotransmission in the nucleus raphe magnus neurons, attenuated by nerve injury and reversed neuropathic allodynia. In mice with SNI, GAD65 downregulation associated with an elevated glutamate/GABA ratio was revealed in the spinal cord [70]. HDAC inhibition restored this imbalance and attenuated mechanical allodynia. SNL induced downregulation of the glutamate transporter type 1 (GLT-1), together with HDAC2 upregulation and histone (H3K9) hypoacetylation in the dorsal horn of rats [71,72]. HDAC inhibition prevented all these alterations. HDAC2 upregulation depended on astrocytic activation (JNK phosphorylation) as an upstream event. Activated microglia contributed to this by increasing TNF-α secretion and consequent JNK phosphorylation in astrocytes. All the above data argue for an epigenetic regulation of the glutamate/GABA balance in the central nervous system by histone deacetylation.

#### 3.4.2. Ion Channels and Transporters

Certain epigenetic mechanisms involve neuron-restrictive silencer factor (NRSF, also known as repressor element 1-silencing transcription factor, REST), which functions as a transcriptional repressor of genes that contain neuron-restrictive silencer element (NRSE, also called response element (RE) 1 [73]. NRSF is a zinc-finger DNA-binding protein, which, upon binding to NRSE, recruits various co-repressor factors such as mSin3 and co-repressor for REST. The formed co-repressor complexes modify target gene regions through HDACs, histone demethylase, etc., leading to a repressive chromatin conformation (heterochromatin). This repressive machinery can be stimulated by interaction with other epigenetic factors, including MeCP2 and Polycomb complexes.

In mice with PSNL, mRNA of K_v_4.3 K^+^ channels that repress neuronal excitability was reduced in DRG neurons [74]. This alteration was associated with a decreased acetylation of H4, but not H3, protein at K_v_4.3-NRSE and an increase in the binding of NRSF to K_v_4.3-NRSE. The nerve injury-evoked K_v_4.3 downregulation was reduced by antisense-knockdown of NRSF. These data suggest that nerve injury enhances NRSF binding and histone hypoacetylation at K_v_4.3-NRSE, leading to K_v_4.3 gene silencing. In the same model, a similar silencing effect, involving increased NRSF expression and binding as well as hypoacetylation at NRSE within the gene of the voltage-gated Na^+^ channel Na_v_1.8 in DRG neurons, was revealed [75,76]. NRSF knockdown blocked nerve injury-induced downregulation of not only Na_v_1.8, but also TRP melastatin 8 (TRPM8) and TRPA1 (though not calcitonin gene-related peptide [CGRP]) in DRG as well, together with C-fiber hypoesthesia, suggesting a role for epigenetic regulation of the negative signs of nerve injury. Interestingly, heat hyperalgesia and mechanical allodynia, underlying the positive signs of nerve injury, were not affected by NRSF knockdown. Various HDAC inhibitors (trichostatin A, valproic acid, and SAHA) restored nerve injury-induced downregulation of Na_v_1.8, TRPM8, and TRPA1 (but not CGRP) in DRG, together with the reversal of decreased C-fiber sensitivity. In addition, trichostatin A reversed the nerve injury-evoked hypoacetylation at H3-/H4-bound Na_v_1.8–NRSE. A similar transcriptional regulation has been revealed in the PSNL model of rats for K_v_7.2/7.3 channels expressed in small-diameter DRG neurons, contributing to the hyperpolarizing M current: nerve injury-increased NRSF expression leads to suppression of the transcription of the K_v_7.2/7.3 genes containing NRSE, which results in neuronal hyperexcitability [77,78].

In rats with CCI, HDAC2 was upregulated in DRG, along with decreased expression of K_v_1.2 channels, co-localizing with HDAC2 in DRG neurons [79]. An intrathecally applied HDAC2 inhibitor or knockdown of HDAC2 by a small interfering RNA (siRNA) treatment reversed K_v_1.2 downregulation and attenuated heat hyperalgesia and mechanical allodynia. These data indicate an epigenetic regulation by histone acetylation of K_v_1.2 channels, contributing to pain hypersensitivity in this model.

In oxaliplatin-treated mice, an NRSF–HDAC3 pathway was revealed that leads to the downregulation of various K^+^ channels in DRG neurons, including two-pore channels such as TREK1 and TRAAK and voltage-gated ones such as Kv1.1 and Kv4.3; this mechanism is involved in the mechanical and cold hypersensitivity [80]. In the neuropathic model based on L5 ventral root transection, the mechanical allodynia was shown to involve upregulation of the Nav1.6 channel in DRG neurons [81]. This response was mediated by the TNF-α-phosphorylated transducer and activator of transcription-3 (pSTAT3)–p300 pathway in the sensory neurons, leading to hyperacetylation of H4, but not H3, histone at the promoter of the Nav1.6 gene.

In a rat model of bone cancer pain, HDAC2 was upregulated in the neurons and astroglia but not the microglia of the spinal cord [82]. In parallel, expression of the K^+^–Cl^−^-cotransporter 2 (KCC2), responsible for maintaining low intracellular Cl^−^ levels, was reduced. Knockdown of HDAC2 restored the reduced expression of KCC2 and reduced mechanical allodynia, whereas trichostatin A suppressed HDAC2 overexpression and promoted H3 acetylation in the spinal cord. The mechanism of the reversal of HDAC upregulation by trichostatin A or other HDAC inhibitors is unclear. Very similar results were obtained in the CCI model of rats: HDAC2 was upregulated in neurons of the ipsilateral spinal cord; HDAC2 knockdown ameliorated mechanical allodynia and heat hyperalgesia, as well as providing increased expression of GAD65 and KCC2; and trichostatin A inhibited hypernociception and decreased elevated HDAC2 levels [83].

#### 3.4.3. Opioid Receptors and Peptides

PSNL enhanced, in mice, NRSF binding to MOP–NRSE in DRG neurons, which reduced MOP receptor gene expression through HDAC recruitment [75]. NRSF knockdown restored both MOP receptor downregulation and morphine antinociception (inhibition of heat hyperalgesia and mechanical allodynia) diminished by nerve injury. In accord, HDAC inhibitors (trichostatin A, valproic acid) restored both the downregulation of MOP receptors and the diminished morphine analgesia upon systemic or local administration [84]. In a model of bone cancer pain in rats, mechanical allodynia was associated with reduced MOP receptor expression in the spinal cord [85]. The nonselective HDAC inhibitor trichostatin A partially reversed both phenomena and potentiated the antinociceptive effect of morphine. In the same model, HDAC1 and 2 proteins were upregulated in DRG, along with the downregulation of MOP receptors [86]. SAHA reduced HDAC expression and increased MOP receptor expression, thereby counteracting morphine tolerance. However, in another study using the same model, the knockdown of HDAC2 failed to restore reduced MOP receptor density [82]. In mice with CCI, HDAC1 upregulation was revealed in the injured DRG, and HDAC1 was shown to bind to the promoter region of the MOP receptor gene [28]. Furthermore, SAHA restored MOP receptor downregulation in DRG and the reduced antinociceptive efficacy of morphine; as well, it prevented the hypoacetylation of the H3 protein.

In rats with CCI to the infraorbital nerve, reduced β-endorphin expression was observed in the hypothalamic arcuate nucleus, along with mechanical allodynia [87]. It has been shown that nerve injury downregulates HDAC9, leading to hyperacetylation (H3K18ac enrichment) of the micro RNA miR-203a-3p gene promoter. This enhances the binding of the transcription factor NR4A2 to the promoter, thereby facilitating miR-203a-3p expression. Upregulated miR-203a-3p reduces the expression of proprotein convertase 1, responsible for the generation of β-endorphin from its precursor proopiomelanocortin.

#### 3.4.4. Cytokines, Chemokines, and Growth Factors

In mice with PSNL, increased acetylation of histone H3 at the promoter region of the chemokine macrophage inflammatory protein (MIP) 2 (a functional murine analog of mammalian IL-8) and its receptor chemokine C-C motif receptor (CXCR) 2 was revealed in the sciatic nerve, and its role in (i) MIP-2 and CXCR2 upregulation, along with IL-1β, TNF-α, MIP-1α, MIP-1β upregulation; (ii) neutrophil and macrophage accumulation in the injured nerve; and (iii) development of tactile allodynia and heat hyperalgesia was demonstrated by using the HAT inhibitor anacardic acid [88]. In the same model, upregulation of C-C motif chemokine ligand (CCL) 2 and CCL3, key players in neuroinflammation associated with neuropathic pain, was revealed in infiltrating macrophages of the injured sciatic nerve, along with an enhanced expression of their receptors (C-C motif chemokine receptor (CCR) 2 and CCR1/CCR5, respectively) [89]. A HAT inhibitor suppressed the upregulation of all these proteins, suggesting that histone acetylation is involved in these responses. Accordingly, euchromatin-promoting H3 histone modifications (increased acetylation and trimethylation) at the CCL2 and CCL3 promoters were revealed in the infiltrating macrophages. In the same model, increased expression of vascular endothelial growth factor (VEGF) A and its vascular endothelial growth factor receptor (VEGFR) 2, but not the VEGFR1 receptor, was detected in the accumulating neutrophils and macrophages of the injured sciatic nerve [90]. At the VEGFA gene promoter, increased H3 acetylation and trimethylation were also revealed in the partially ligated sciatic nerve. A contribution of both VEGFA and its receptor to the nerve injury-induced tactile allodynia and heat hyperalgesia was also demonstrated. Also, in the same model, a significant contribution of BDNF upregulation in DRG and spinal dorsal horn to injury-evoked heat hyperalgesia and mechanical allodynia was revealed at an early stage on day 2 [91]. It was also shown that histone H3 and H4 acetylation was increased at the BDNF gene promoter, providing evidence for the epigenetic control of BDNF expression in this model of neuropathic pain.

In the rat CCI model, functional, synaptically located DOP receptors are induced in the brainstem nucleus raphe magnus by pharmacological inhibition of HDAC [92]. Its mechanism involves hyperacetylation of the H4 protein at the nerve growth factor (NGF) gene promoter region, leading to increased NGF formation, with consequent DOP receptor upregulation responsible for the mechanical antiallodynic effect.

In the case of the paclitaxel-induced mechanical allodynia, the following signaling pathway has been revealed in rats: systemic paclitaxel treatment upregulates the phosphorylated p65 subunit of NF-κB in the neurons of the dorsal horn, which binds to the promoter region of the chemokine C-X3-C motif ligand (CX3CL) 1 gene and increases acetylation of H4, but not H3, at the promoter, leading to enhanced expression of the CX3CL1 in spinal neurons [93]. Furthermore, paclitaxel administration also upregulates C-X-C motif ligand (CXCL) 12 expression in the dorsal horn via a mechanism involving signal transducer and activator (STAT) 3 activation by phosphorylation, pSTAT3 binding to the promoter region of the CXCL12 gene, and recruitment of the HAT enzyme p300, leading to increased acetylation of H4, but not H3, in the CXCL12 promoter [94]. In mice with CCI, SNL, or SNI, expression of a zinc-finger protein (ZNF)-type transcription factor ZNF382 is decreased in injured DRG neurons but not the spinal cord [95]. Normally, ZNF382 forms a trimeric complex in which HDAC1 and the histone methyltransferase (HMT) SET domain bifurcated histone lysine methyltransferase 1 (SETDB1) are bound to the promoter of the CXCL13 gene, leading to transcriptional repression via reduced H3 acetylation and increased H3K9me3 methylation. Nerve injury-evoked ZNF382 downregulation leads to disinhibition of CXCL13 gene transcription and, thus, CXCL13 upregulation, which contributes to mechanical allodynia and heat hyperalgesia.

In spontaneously diabetic (type 2, Zucker) mice and rats, expression of the proinflammatory cytokine high-mobility group box (HMGB) 1 was increased in microglia cells in the spinal cord [96]. This was due to enhanced histone acetylation (H3K9) at the promoter of the HMGB1 gene. HMGB1 upregulation was associated with JNK activation and increased expression of toll-like receptor 4 (TLR4), CXCR4 receptor, and NLRP3, as well as mechanical and heat hyperalgesia.

In a rat model of bone cancer pain, HDAC1 was upregulated mainly in neurons and microglia of the spinal dorsal horn, while HDAC2 was upregulated in spinal astrocytes; both enzymes were upregulated in satellite glial cells of the DRG [47]. Tumor cell-induced HDAC upregulation increased secretion of TNF-α, IL-1β, and IL-6, in both the dorsal horn and DRG. Expression of glycogen synthase kinase-3 regulator of neuroinflammation decreased in the dorsal horn and DRG. SAHA administration reversed all the above changes, as well as mechanical allodynia.

In rats with brachial plexus avulsion, an intricate signaling pathway of mechanical allodynia with an epigenetic control has been identified [97]. In this neuropathic model, decreased histone acetylation (at H3K9 and histone H4 at lysine (H4K) 12) as a result of HDAC activation could be verified in the DRG. This led to the activation of phosphatidylinositol 3-kinase, which can phosphorylate and thereby activate Akt (also known as protein kinase B). Downstream elements of Akt in this model include activation of mTOR involved in IL-2 signal transduction, formation of proinflammatory cytokines such as TNF-α, IL-1β, and IL-6, as well as increased expression of TR vanilloid 1 (TRPV1) and TRPM8 ion channels.

In mice with spinal cord injury, inhibition by JQ1 of BET proteins, recognizing acetylated lysine residues in histone proteins, reduced the formation of proinflammatory cytokines (e.g., TNF-α, IL-1β, IL-6), along with an increase in anti-inflammatory cytokine (e.g., IL-4, IL-10, IL-13) production in the spinal cord [98]. BET inhibition also reduced mechanical allodynia, suggesting that, in this model, BET proteins might contribute to injury-evoked hypernociception via altered cytokine production in the spinal cord.

#### 3.4.5. Inflammasome Proteins

Repeated systemic administration of the proteasome inhibitor bortezomib evoked an increase in the level of pSTAT3 [99]. Bortezomib also induced STAT3 recruitment to the promoter of the gene of NLRP3, a core inflammasome component, in DRG, along with increased acetylation of H3 and H4 in the NLRP3 promoter, suggesting that STAT3 upregulated NLRP3 expression through increased histone acetylation. NLRP3 knockdown by siRNA attenuated bortezomib-evoked mechanical allodynia, whereas overexpression of NLRP3 in DRG reduced the mechanonociceptive threshold in naive rats, showing the role of NLRP3 upregulation in mechanical allodynia. In the same model, bortezomib administration decreased the expression of SIRT1, whereas it enhanced the expression of NACHT leucine-rich-repeat protein (NALP) 1, an inflammasome-forming protein, in the dorsal horn [62]. Both SIRT1 deficiency and NALP1 upregulation were involved in the mechanical allodynia. It was also shown that SIRT1 reduction increased the phosphorylation of STAT3, which led to NALP1 upregulation by pSTAT3-mediated histone H3 and H4 acetylation at the promoter region of the NALP1 gene. In the latter effect, enhanced interaction between pSTAT3 and the HAT enzyme p300 was implicated.

#### 3.4.6. Other Proteins

In rats with CCI, overexpression of the cyclooxygenase 2 (COX-2) enzyme, along with upregulation of the HAT p300 in the dorsal horn, was found [100]. Intrathecal siRNA treatment against p300 decreased COX-2 expression as well as nerve injury-evoked mechanical allodynia and heat hyperalgesia. In neuropathic animals, an enhanced p300 binding to the promoter region of the COX-2 gene was also demonstrated, showing that nerve injury leads to COX-2 upregulation through histone acetylation by p300. In the same model, the upregulation of cyclin-dependent kinase 5 (Cdk5) in the dorsal horn contributed to nerve injury-induced tactile allodynia [101]. Nerve injury increased the expression and binding of phosphorylated CREB to the Cdk5 gene promoter and enhanced histone H4 acetylation there. As CREB has HAT activity, it was most likel involved in the increased H4 acetylation.

In mice with PSNL, HDAC5 upregulation in neurons and glial cells of the spinal cord led to mechanical allodynia and heat hyperalgesia [102]. HDAC5 was shown to bind to the promoter region of the transcription factor SOX10 gene and, thereby, increase SOX10 expression. In mice with SNL, the transcription factor-specific protein 1 (Sp1) was upregulated in the spinal dorsal horn, which enhanced the expression of HDAC1 by binding to its promoter [103]. HDAC1, in turn, increased the expression of SOX10 by binding to its promoter, leading to mechanical allodynia and heat hyperalgesia. CCI injury in rats also induced upregulation of Sp1 in the spinal cord [104]. In this model, Sp1 was shown to bind to HDAC2, which then promoted histone deacetylation at the promoter of the peroxisome proliferator-activated receptor-γ coactivator-1α (PGC-1α) gene, resulting in PGC-1α downregulation and consequent aggravation of mitochondrial dysfunction and oxidative stress, leading to mechanical allodynia and heat hyperalgesia.

In DRGs of rats with SNL, a diminished HDAC2 occupancy at the promoter of the Ca^2+^ channel subunit α2δ-1 gene led to histone hyperacetylation of the α2δ-1 gene [105]. This increased the expression of α2δ-1, which maintains neuropathic pain by recruiting N-methyl-D-aspartate (NMDA) receptors from the cytoplasm to the presynaptic and postsynaptic membrane (independently of voltage-gated Ca^2+^ channels), thus facilitating central sensitization in the dorsal horn. In naive rats, the knockdown of HDAC2, but not HDAC3, reduced the baseline mechanonociceptive but not the noxious heat threshold, in agreement with the proposed tonic inhibitory effect of HDAC2 on α2δ-1 expression and consequent mechanical allodynia. Bortezomib treatment upregulated the transcription factor GATA3, which inhibited transcription of the gene for the AMP-activated kinase (AMPK) a2 by enhanced H3 methylation and reduced H3 acetylation at the promoter [106]. AMPKa2 downregulation led to diminished expression of Beclin-I, which is a key autophagy marker. All these changes were reversed by metformin, an AMPK activator. Beclin-I downregulation in the dorsal horn also contributes to oxaliplatin-induced neuropathic mechanical allodynia, which involves upregulation of the transcription factor nuclear factor of activated T cells type 2 (NFATc2) in the rat spinal dorsal horn [107]. NFATc2 reduced the expression of the autophagy-related factor tuberous sclerosis complex protein 2 (TSC2) by enhanced H4 methylation at the promoter of the TSC2 gene, with consequent reduction of H4 acetylation. TSC2 downregulation reduces autophagy by decreasing Beclin-I expression, which contributes to neuropathic pain.

Epigenetically relevant details of the neuropathy models in which histone acetylation was investigated are found in Table 2.

## 4. Histone Protein Modification by Methylation/Demethylation in Neuropathic Conditions

### 4.1. General Principles

A less extensively studied form of posttranslational histone modification is methylation, which occurs on the tail of histone proteins at lysine or arginine residues [44,108]. Lysine can be mono-, di-, or trimethylated, whereas arginine can be mono- or dimethylated. The enzymes responsible for these modifications are HMTs. Dimethylation of histone H3 at lysine 9 residue (yielding H3K9me2) requires lysine dimethyltransferase G9a and G9a-like protein (GLP), which form a heterodimer (G9a/GLP). The H3K9me3-specific histone methyltransferase is the suppressor of variegation 3-9 homolog 1 (SUV39H1). The trimethylation of H3 at lysine 27 (resulting in H3K27me3) depends on the enhancer of the HMT zeste homolog-2 (EZH2). Histone methylation can lead to either transcriptional activation (e.g., H3K4me2, H3K4me3, H3K79me3) or repression (e.g., H3K9me2, H3K27me3). Methylation of an arginine residue can be performed by nine protein arginine methyltransferase (PRMT) enzymes (PRMT1–3, PRMT4/coactivator-associated arginine methyltransferase (CARM) 1, PRMT5–8, PRMT9/10). Of these, CARM1/PRMT4 catalyzes dimethylation on Arg17 and 26 of the H3 protein (yielding H3R17me2 and H3R26me2, respectively), leading to transcriptional activation. Methyl groups are removed by histone lysine demethylase (KDM) enzymes (KDM1–6) and arginine demethylases, including, among others, jumonji domain-containing protein (JMJD) 6 and peptidyl arginine deiminase (PADI) 4. All nine PRMT and the two arginine demethylase JMJD6 and PADI4 enzymes were shown to be expressed in the DRG [109].

### 4.2. Proteins Regulated by Histone Methylation/Demethylation

#### 4.2.1. K^+^ Channels

In a seminal study on rats with SNL, the epigenetic regulation of four K^+^ channel types involved in neuropathic pain was investigated: three voltage-gated K^+^ channels, including K_v_1.4, K_v_4.2, and K_v_7.2, as well as large-conductance Ca^2+^-activated K^+^ channels [45]. SNL diminished mRNA levels in all these channels but upregulated the HMT enzymes G9a and EZH2 in DRG neurons. In accordance, nerve injury increased H3K9me2 and H3K27me3 levels in the DRG but failed to alter G9a and H3K9me2 expression in the spinal cord. SNL increased H3K9me2 occupancy at the promoters of all four K^+^ channel genes but failed to affect H3K27me3 enrichment in any of these genes. No change in the DNA methylation status at the promoter of the four K^+^ channel genes was observed. Using a specific inhibitor or knockdown of G9a, it was shown that G9a plays a dominant role in enhancing the H3K9me2 protein level and decreasing the mRNA levels in the four K^+^ channel types. In addition, G9a was shown to attenuate K_v_ currents in DRG neurons and the development of mechanical hyperalgesia and tactile allodynia. Furthermore, the genome-wide analysis revealed that the expression of 42 K^+^ channel genes was downregulated by SNL; of them, 40 were partially normalized by a G9a inhibitor. Similarly, 2035 genes were identified whose expression levels were altered by nerve injury at least 2-fold. Of these, the expressions of 396 were partially normalized, and 242 were completely restored by a G9a inhibitor. These data indicate the fundamental role of histone methylation by G9a in the epigenetic control of K^+^ channel expression in the SNL model. In a subsequent study on mice with SNL, consonant results were obtained, in that the expression of G9a was increased in both peptidergic and non-peptidergic neurons but not glial cells of DRG, along with an increase in the level of H3K9me2 [110]. G9a upregulation was also evident in the CCI model, but not after complete Freund’s adjuvant treatment. G9a upregulation was shown to contribute to SNL-induced mechanical allodynia and heat or cold hyperalgesia via reduced expression of the K_v_1.2 protein, which leads to the diminishment of total K_v_ current and an increase in excitability of DRG neurons (by enhancing the resting potential, reducing the current threshold, and increasing the number of action potentials). Some K^+^ channels are under epigenetic regulation by histone arginine methyltransferase enzymes. CARM1 was upregulated in the nuclei of injured DRG neurons following nerve injury (SNL or CCI) in mice, whereas the arginine demethylase (JMJD6 and PADI4) expression remained unaltered [109]. CARM1 upregulation was required for neuropathic mechanical allodynia and heat hyperalgesia. Contrasting results were obtained in rats with SNL, as downregulation of CARM1 in the dorsal horn neurons was responsible for the mechanical allodynia. This was associated with a decreased H3R17me2 level at the promoter of the genes of the K_v_1.4 and K_v_4.2 channels, leading to the downregulation of these channels, explaining neuropathic hypernociception [111].

#### 4.2.2. Opioid Receptors

G9a is also involved in the epigenetic control of opioid receptor expression under neuropathic conditions. In rats or mice with SNL, a downregulation of MOP, DOP, and KOP receptors in the affected DRG (but not the dorsal horn) was observed, along with reduced antinociceptive actions of applied opioids [112,113]. Nerve injury upregulated G9a at the injured DRG, increased the H3K9me2 level at the promoter region of the MOP receptor gene, and reduced CREB binding to its motif. Inhibition of G9a by a specific inhibitor or siRNA or conditional genetic ablation prevented opioid receptor downregulation and preserved opioid analgesia, providing evidence for the role of G9a-mediated histone methylation, leading to reduced CREB binding in this model.

#### 4.2.3. Other Proteins

Expression of EZH2 and its product H3K27me3 has been revealed in rat neurons and microglia of the dorsal horn, with strong upregulation by PSNL in neurons and even more in microglia [114]. EZH2 overexpression led to enhanced formation of IL-1β and TNF-α in the dorsal horn, along with mechanical allodynia and heat hyperalgesia. In rats, CCI enhanced the CGRP expression in the dorsal horn, which led to EZH2 upregulation and H3K27me3 enrichment in microglia through a protein kinase A (PKA)-/protein kinase C (PKC)-mediated pathway [115]. In addition, in a glial cell line, applied CGRP altered H3K27me3 enrichments at the promoters of 248 genes (173 increased, 75 decreased), several among them associated with microglial activation. Novel histone methylation modifications were revealed in microglia isolated from the brains of cisplatin-treated mice: H3.1K27me was enriched and H3K56me3 was decreased compared to untreated mice [116]. Reversal of these downregulations by a selective KDM7A inhibitor attenuated mechanical allodynia in the neuropathic animals. Bortezomib-induced mechanical allodynia was associated with an increased expression of KDM6 in the spinal cord, along with upregulation of peroxisome proliferator-activated receptor (PPAR), IL-6, prokineticin 2, and IL-1β [117]. As a prokineticin receptor antagonist prevented KDM6 upregulation, prokineticins can control the expression of histone demethylase (HDM) enzymes in this model. In mice with SNI or CCI, macrophage migration-inhibiting factor (MIF) was overexpressed in the ventral tegmental area and evoked both G9a/GLP and SUV39H1 upregulation, along with enhanced DNA (CpG island) methylation in the tyrosine hydroxylase gene [118,119]. This led to the downregulation of tyrosine hydroxylase and dopamine levels, resulting in mechanical allodynia and heat hyperalgesia by inhibiting the descending antinociceptive dopaminergic pathway(s). Increased DNA methylation of the tyrosine hydroxylase gene could be due to DNMT3a and DNMT1 recruitment directly by G9a or via previous H3K9me2 formation [108,120].

SNL-evoked mechanical allodynia and heat hyperalgesia in the rat involved an increased IL-6 expression in the affected DRG and dorsal horn [121]. IL-6 upregulation was due to enhanced histone demethylation by KDM6B, an HDAC enzyme specific for removing H3K27me3 marks at gene promoters. This demethylation induced binding of p-STAT3 to the promoter of the IL-6 gene. KDM6B expression was revealed in both peptidergic and non-peptidergic neurons of the DRG and the dorsal horn and was upregulated by nerve injury. In the same model, cannabinoid 2 (CB_2_) receptors were upregulated in DRG neurons [122]. This alteration was associated with increased enrichment of two activating histone marks (H3K4me3 and H3K9ac) and reduced enrichment of two repressing histone marks (H3K9me2 and H3K27me3) at the promoter of the CB_2_ receptor gene. In contrast, the DNA methylation status of the CB_2_ cannabinoid receptor gene promoter remained unchanged. As shown in a recent study, paclitaxel-induced mechanical allodynia and heat hyperalgesia involved the upregulation, in DRG neurons, of phosphorylated p90 ribosomal S6 kinase 2 (pRSK2), which activated (by phosphorylation) a NIMA-related kinase (NEK) 2, regulating microtubule dynamics [123]. pNEK2 activated KDM6B, which removed H3K27me3 marks at the promoter of the TRPV1 receptor gene, resulting in enhanced TRPV1 expression in DRG neurons. In the same neuropathy model, a complementary mechanism has been revealed [124]. Paclitaxel upregulated PRMT5 in DRG neurons, which increased H3R2me2 enrichment at the promoter of the TRPV1 gene. WD repeat-containing protein (WDR) 5, as an epigenetic reader protein, interacts with H3R2me2 and enhances H3K4me3 enrichment, leading to transcriptional activation of TRPV1. In the SNL and SNI models of rats, the upregulation of the H3K4me3-specific HMT, called mixed lineage leukemia type 1 (MLL1), in dorsal horn neurons and its interaction with WDR5 leads to H3K4me3 enrichment at the promoter of the mGlu5 receptor gene, resulting in transcriptional activation [125].

In mice with PSNL, expression of monocyte chemotactic protein (MCP) 3 (also known as CCL7) was drastically increased in the astrocytes of the ipsilateral spinal cord, along with a decreased trimethylation of H3 at Lys27 at the MCP-3 gene promoter [126]. Both alterations were almost abolished in IL-6 gene-deficient mice with nerve injury. Evidence has been provided for an IL-6–pSTAT3/PU.1–H3K27me3 pathway for the nerve injury-induced MCP-3 upregulation. The latter alteration could contribute to nerve injury-evoked heat hyperalgesia and mechanical allodynia via activation of CCR2 receptors on microglia.

Epigenetically relevant details of the neuropathy models in which DNA methylation was investigated are shown in Table 3.

## 5. Epigenetic Regulation of the Baseline Noxious Mechanical or Heat Threshold

Relatively little data are available regarding the epigenetic regulation of nociceptive responsiveness under normal conditions, i.e., without inflammation or nerve injury. Relevant to DNA methylation, a genome-wide human study that enrolled 50 monozygotic twins revealed an association between decreased heat pain sensitivity and TRPA1 gene-promoter hypermethylation and decreased TRPA1 mRNA levels in blood cells [127]. Repeated intrathecal injection of a DNMT inhibitor caused mechanical hypersensitivity in naive rats [15]. Overexpression of DNMT3a in DRG evoked mechanical allodynia and heat and cold hyperalgesia in uninjured rats and mice [6]. Microinjection of recombinant OCT1 to the lumbar DRGs evoked mechanical allodynia, heat hyperalgesia, and cold allodynia in naive rats [29]. MBD1 knockout mice exhibited reduced responses to noxious mechanical, heat, cold, and chemical (capsaicin) stimuli in uninjured animals [10]. It was shown that MBD1 inhibits the basal expression of MOP and KOP (but not DOP) receptors and K_v_1.2 channels in the DRG of naive mice. Similar changes were observed in mice with siRNA-induced knockdown of MBD1 in DRG. Overexpression of MBD1 in DRG in naive mice led to increased responsiveness to noxious heat, cold, and mechanical stimuli. Intrathecal TET1 gene transfer by a viral vector resulted in mechanical allodynia and heat hyperalgesia in naive rats [35]. This treatment enhanced TET1 expression as well as TET1-mediated hypomethylation of the mGlu5 receptor gene promoter, causing increased expression of this receptor. Knockdown of TET1 expression by siRNA-targeting TET1 delivered intrathecally prevented all the above changes. Contradictory results were obtained in another study [31], showing that viral delivery of TET1 mRNA to the L5 DRG of naive rats failed to evoke alteration of basal heat or mechanical pain sensitivity. In naive rats, overexpression of DNMT3a in the dorsal horn reduced K_v_1.2 channel expression and induced mechanical allodynia, heat hyperalgesia, and cold allodynia [8]. Mice with a loss of function mutation of MeCP2 have decreased mechanical pain sensitivity [33].

Regarding histone acetylation, in mice expressing a modified form of HDAC4 that lacks the putative C-terminal catalytic domain, an impaired thermonociception was revealed by hot plate assay [128]. In these animals, the formalin-evoked nociception was not altered in either phase. Knockdown of SIRT1 induced heat hyperalgesia, mechanical allodynia, and c-fos activation in the spinal cord of naive rats [48]. A non-selective HDA inhibitor (quisinostat) evoked mechanical hyperalgesia in naive mice [129]. In naive rats, genetic downregulation of HDAC2, but not HDAC3, reduced the baseline mechanonociceptive but not the noxious heat threshold [95].

Relevant for histone methylation, overexpression of G9a evoked mechanical allodynia and heat or cold hyperalgesia in intact mice and reduced expression of K_v_1.2 channel protein, whereas knockdown of G9a increased the levels of K_v_1.2 channels [110]. In naive rats, knockdown or pharmacological inhibition of CARM1 induced mechanical allodynia through decreased H3R17me2 level at the promoter of the genes of the K_v_1.4 and K_v_1.2 channels in the dorsal horn [111].

## 6. Conclusions

Ample experimental evidence supports the view that several molecular targets playing prominent roles in the generation of neuropathic pain are under epigenetic control. DNA methylation, histone acetylation, and methylation are the most frequent covalent chemical alterations of the chromatin that can modify gene expression at a transcriptional level. Downregulation of opioid receptors and several types of voltage-gated K^+^ channels and upregulation of certain glutamate receptors, growth factors, and lymphokines have been shown to be evoked by the above epigenetic modifications. Studies have also identified several epigenetic writer, reader, and eraser proteins that are responsible for the formation, function, and removal of the covalently bound epigenetic marks. A better understanding of the functional significance of the epigenetic alterations may help identify target structures for the development of novel drugs against neuropathic pain. As a prerequisite for this, various inhibitors of the key enzymes of the epigenetic machinery (DNMT, TET, HAT, HDAC, HMT, KDM, JMDJ), called epigenetic drugs, are available [130,131]. Many of them are already in clinical development or even used for human diseases—first of all, cancer. Repurposing of these agents for the treatment of neuropathic pain is faster and more cost-effective than the development of novel compounds screened so far only in preclinical studies. Investigation of these epigenetic drugs has revealed additive interactions between drugs altering DNA methylation and histone modifications. However, epigenetic drug development is not an easy task, as the target enzymes have multiple isoforms, making the development of subtype-specific inhibitors difficult. Table 4 summarizes the epigenetic enzyme inhibitors tested so far in various animal models of neuropathic pain. This table provides an overview of the pharmacological modulation possibilities of the epigenetic machinery; thus, it can pave the way for the development of novel epigenetic drugs that target neuropathic pain.

## Figures and Tables

**Table 1:** Experimental models in which the effects of DNA methylation were examined.

Neuropathy Model	Species	Epigenetic Alteration(s)	Epigenetically Regulated Change(s) in Gene Expression	Epigenetically Regulated Nociceptive Response(s)	Reference(s)
Chronic constriction injury (CCI)	Male Sprague–Dawley rats	Global DNA hypermethylation, MeCP2 mRNA, and nuclear MeCP2 protein upregulation in the spinal cord	–	Mechanical allodynia, heat hyperalgesia	[9]
	Male C57BL/6J mice	Hypermethylation of the MOP gene proximal promoter	MOP downregulation in DRG and spinal cord	Heat hyperalgesia	[27]
	Male C57BL/6J mice	Hypermethylation + increased MeCP2 binding at the MOP gene promoter	MOP downregulation in DRG neurons	Heat hyperalgesia	[28]
	Male C57BL/6J mice	DNMT3a expression ↑ → methylation of MOP gene promoter ↑	MOP downregulation in the spinal cord	Heat hyperalgesia	[30]
	Male Sprague–Dawley rats	OCT1 upregulation → DNMT3a upregulation	MOP and K_v_1.2 downregulation in DRG neurons	Mechanical allodynia, heat hyperalgesia, cold allodynia	[29]
	Male C57BL/6J mice	CREB upregulation → DNMT1 upregulation	K_v_1.2 downregulation in DRG neurons	Mechanical allodynia, heat hyperalgesia, cold allodynia	[7]
	Male Sprague–Dawley rats	DNMT3a, 3b, MeCP2 upregulation → hypermethylation at the promoter of the GAD67 gene	GAD67 mRNA downregulation in the spinal cord	Mechanical allodynia	[34]
	Male Sprague–Dawley rats	Hypermethylation of the mGlu4, 5-HT_4_, β_2_, K_v_5.1 genes	mGlu4, 5-HT_4_, β_2_, K_v_5.1 mRNA decrease in the spinal cord	Mechanical allodynia, heat hyperalgesia	[13]
Spinal nerve ligation (SNL)	Male Sprague–Dawley rats; C57B/L6 mice	DNMT3a upregulation → hypermethylation of the MOP gene promoter + increased MBD1 binding	MOP and KOP downregulation in DRG	Heat hyperalgesia	[5]
	Male Sprague–Dawley rats	OCT1 upregulation → DNMT3a upregulation → hypermethylation at the K_v_1.2 gene promoter	K_v_1.2 downregulation in DRG neurons	Mechanical allodynia, heat, and cold hyperalgesia	[6]
	Male C57BL/6J mice	CREB upregulation → DNMT1 upregulation	K_v_1.2 downregulation in DRG neurons	Mechanical allodynia, heat hyperalgesia, cold allodynia	[7]
	Male C57BL/6J mice	MBD1 upregulation → DNMT3a binding to MOP and K_v_1.2 gene promoter ↑	MOP and K_v_1.2 downregulation in DRG neurons	Mechanical allodynia, heat hyperalgesia, cold allodynia	[10]
	Male Sprague–Dawley rats	DNA hypermethylation at the promoters of the MOP and K_v_1.2 genes	MOP and K_v_1.2 downregulation in DRG	Mechanical allodynia, heat hyperalgesia	[31]
	Male Sprague–Dawley rats	TET1 upregulation, TET1–mGlu5 coupling → demethylation of the mGlu5 gene promoter ↑	mGlu5 upregulation in the dorsal horn	Mechanical allodynia, heat hyperalgesia	[35]
	Male Sprague–Dawley rats	DNMT1, 3a, 3b binding to the BDNF gene promoter ↑, methylation ↑, later TET1 upregulation → demethylation at the CpG sites of the BDNF gene promoter	BDNF upregulation in the dorsal horn	Mechanical allodynia	[12]
	Male Sprague–Dawley rats	CpG hypomethylation in DRG → hypermethylation in the spinal cord and prefrontal cortex	1684 genes are upregulated, 1039 genes are downregulated in DRG	Mechanical allodynia	[15]
	Male Sprague–Dawley rats	Hypermethylation of the mGlu4, 5-HT_4_, β_2_, K_v_5.1 genes	mGlu4, 5-HT_4_, β_2_, K_v_5.1 mRNA decrease in the spinal cord	Mechanical allodynia, heat hyperalgesia	[13]
Spared nerve injury (SNI)	Male CD1 mice	Global DNA hypomethylation in the prefrontal cortex	–	Mechanical allodynia, cold hyperalgesia	[17]
	Male Sprague–Dawley rats	Global DNA hypomethylation in the prefrontal cortex	–	Mechanical allodynia	[18]
	Male Sprague–Dawley rats	TET1 expression in the prefrontal cortex ↑	–	–	[22]
	Male Sprague–Dawley rats	DNMT1 expression in the hippocampus ↑	–	–	[21]
	Male Sprague–Dawley rats	mRNAs of DNMT1, 3a, 3b and MeCP2 ↓ in the dorsal horn	–	–	[4]
	Male C57BL/6J mice	TET3 mRNA ↑ → DNA hypomethylation in DRG neurons	–	–	[11]
	Male C57BL/6J mice	MeCP2 upregulation in DRG	BDNF upregulation in DRG	Mechanical allodynia, heat hyperalgesia	[33]
Partial sciatic nerve ligation (PSNL)	Male C57BL/6J mice	Global DNA hypomethylation in the prefrontal cortex + DNMT1, 3a, 3b expression ↓	–	Mechanical allodynia, cold allodynia	[23]
Axotomy	Male C57BL/6J mice	CREB upregulation → DNMT1 upregulation	K_v_1.2 downregulation in DRG neurons	Mechanical allodynia, heat hyperalgesia, cold allodynia	[7]
Tibial nerve transection	Male C57BL/6 mice	Global DNA hypermethylation in the primary somatosensory cortex	–	–	[24]
Cancer pain	Human cancer specimen + female BALB/c mice	Hypermethylation at the promoter of the ET_B_ gene	Downregulation of the ET_B_ mRNA	Mechanical allodynia	[38]
	Male Copenhagen rats	DNMT3a upregulation	K_v_1.2 downregulation in the dorsal horn	Mechanical allodynia, heat hyperalgesia, cold allodynia	[8]
Cancer chemotherapy-induced neuropathic pain	Male CD1 mice	Paclitaxel-induced DNMT3a upregulation and binding → hypermethylation at the K_2p_1.1 promoter	K_2p_1.1 downregulation in DRG neurons	Mechanical allodynia, heat hyperalgesia	[32]
	Male Sprague–Dawley rats	Oxaliplatin-induced TET1 upregulation → demethylation of the SOX10 gene promoter	SOX10 upregulation → HOXA6 upregulation in dorsal horn neurons	Mechanical allodynia	[39]
	Male Sprague–Dawley rats (oxaliplatin)	ZEB1, DNMT3b upregulation → hypermethylation at the promoter of the DDR1 gene	DDR1 downregulation in the dorsal horn neurons	Mechanical allodynia	[40]
Diabetic neuropathy	Female Sprague–Dawley rats	DNMT3b downregulation in DRG → hypomethylation of CpG sites at the promoter of the P2X3 gene	P2X3 upregulation in DRG	Mechanical allodynia, heat hyperalgesia	[36]
	C57BL/6 mice	TET2 upregulation → hypomethylation in the TXNIP gene	TXNIP upregulation in DRG neurons	Mechanical allodynia, heat hyperalgesia	[37]
Chronic low back pain	Female C57BL/6J mice + Humans	Hypermethylation of the SPARC gene promoter	Downregulation of SPARC in intervertebral discs	Increased axial pain	[41]
Chronic pain	Patients	DNA hypermethylation at the CpG island of the TRPA1 gene	TRPA1 mRNA in blood cells ↓	DN4 questionnaire score ↑	[42]

The upward and downward arrows mean increases and decreases of the factor or process, respectively. The horizontal arrows indicate consequences.

**Table 2:** Experimental models in which the effects of histone acetylation were examined.

Neuropathy Model	Species	Epigenetic Alteration(s)	Epigenetically Regulated Change(s) in Gene Expression	Epigenetically Regulated Nociceptive Response(s)	Reference(s)
Partial sciatic nerve ligation (PSNL)	Male Wistar rats	Histone acetylation in H3K9 promoters ↓ in spinal cord	–	Mechanical allodynia, heat hyperalgesia	[50]
	Male C57BL/6J mice	H4 acetylation at K_v_4.3-NRSE ↓	K_v_4.3 mRNA in DRG ↓	–	[74]
	Male C57BL/6J mice	H3/H4 hypoacetylation at NRSEs within MOP and Na_v_1.8 genes ↓	NRSF upregulation in DRG → MOP, Na_v_1.8, TRPM8, TRPA1 expression in the DRG ↓	Tactile allodynia, heat hyperalgesia	[75,84]
	Male ICR mice	H3 hyperacetylation at the MIP-2 and CXCR2 promoter in the sciatic nerve	Upregulation of MIP-2, CXCR2, IL-1β, TNF-α, MIP-1α, MIP-1β in neutrophils, macrophages	Tactile allodynia, heat hyperalgesia	[88]
	Male ICR mice	H3 hyperacetylation at the CCL2 and CCL3 gene promoters in the sciatic nerve	mRNA of CCL2, CCL3, CCR2, CCR1/5 ↑ in macrophages	–	[89]
	Male IRC mice	H3 hyperacetylation at the VEGF gene promoter in the sciatic nerve	VEGFA upregulation in macrophages, neutrophils	–	[90]
	Male C57BL/6J mice	H3 and H4 hyperacetylation at BDNF gene promoter	BDNF upregulation in the dorsal horn	–	[91]
	Male C57BL/6J mice	HDAC1 upregulation, H3K9 hypoacetylation in microglia of the dorsal horn	–	–	[49]
	Male Wistar rats	REST upregulation in DRG	K_v_7.2, K_v_ 7.3 mRNA downregulation in DRG	Mechanical allodynia, heat hyperalgesia	[77,78]
	Male C57BL/6J mice	HDAC5 upregulation at the promoter of the SOX10 gene	SOX10 upregulation in dorsal horn neurons	Mechanical allodynia, heat hyperalgesia	[102]
	Male C57BL/6J mice	H3 and H4 hypoacetylation at Na_v_1.8–NRSE	Na_v_1.8 mRNA ↓ TRPA1 mRNA ↓ TRPM8 mRNA ↓ in DRG	C-fiber hypoesthesia	[76]
Chronic constriction injury (CCI)	Male Sprague–Dawley rats	p300 upregulation and recruitment to the COX-2 gene promoter	COX-2 upregulation in the dorsal horn	Mechanical allodynia, heat hyperalgesia	[100]
	Male Sprague–Dawley rats	SIRT1 downregulation → H3 hyperacetylation in the spinal cord	–	Mechanical allodynia, heat hyperalgesia	[60]
	Male Kunming mice	SIRT1 downregulation → H4 hyperacetylation in the spinal cord	–	Mechanical allodynia, heat hyperalgesia	[61]
	Male Sprague–Dawley rats	HDAC overactivity	Upregulation of mTOR, NF-κB, IL-6, TNF-α, iNOS	Mechanical allodynia, heat hyperalgesia	[63]
	Male Sprague–Dawley rats	phosphorylated CREB → H4 hyperacetylation at the Cdk5 gene promoter	Cdk5 mRNA ↑ in the dorsal horn	Mechanical allodynia, heat hyperalgesia	[101]
	Male Sprague–Dawley rats	HDAC2 upregulation	Downregulation of GAD65 and KCC2 in the spinal cord	Mechanical allodynia, heat hyperalgesia	[83]
	Male Sprague–Dawley rats	HDAC2 upregulation	K_v_1.2 downregulation in DRG neurons	Mechanical allodynia, heat hyperalgesia	[79]
	Male C57BL/6 mice	HDAC1 upregulation, reduced H3 acetylation at the MOP gene promoter in DRG	Decreased MOP expression in the DRG	Heat hyperalgesia	[28]
	Male C57BL/6J mice	H3 hyperacetylation at the CXCL13 promoter	CXCL13 upregulation in DRG neurons	Mechanical allodynia, heat hyperalgesia	[95]
	Sprague–Dawley rats	Sp1 ↑ → HDAC2 binding to PGC-1α gene promoter	PGC-1α downregulation in the spinal cord	Mechanical allodynia, heat hyperalgesia	[104]
	Male KM mice	ETSI ↑ → HDAC1 upregulation in DRG neurons	–	Mechanical allodynia, heat hyperalgesia	[64]
Spinal nerve ligation (SNL)	Male Wistar rats	Histone acetylation in H3K9 promoters ↓ in spinal cord	–	Mechanical allodynia, heat hyperalgesia	[50]
	Male Wistar rats	HDAC1 upregulation, H3 hypoacetylation in the dorsal horn	–	Mechanical allodynia, heat hyperalgesia	[51]
	Male Wistar rats	H3 hypoacetylation at the promoter of GAD65 gene	GAD65 downregulation in the nucleus raphe magnus	Mechanical allodynia	[69]
	Male Sprague–Dawley rats	Hypoacetylation of H3K9	GLT-1 downregulation in the dorsal horn	Mechanical allodynia, heat hyperalgesia	[71]
	Male Sprague–Dawley rats	TNF-α + phosphorylated JNK → HDAC2 upregulation	GLT-1 downregulation in the dorsal horn	Mechanical allodynia	[72]
	Male C57BL/6J mice	H3 hyperacetylation at the CXCL13 promoter	CXCL13 upregulation in DRG neurons	Mechanical allodynia, heat hyperalgesia	[95]
	Male C57BL/6J mice	Sp1 ↑ → HDAC1 upregulation and binding to the SOX10 gene promoter	SOX10 upregulation in the dorsal horn	Mechanical allodynia, heat hyperalgesia	[103]
	Male Sprague–Dawley rats	HDAC2 occupancy at the α2δ-1 gene promoter ↓ → hyperacetylation	α2δ-1 upregulation in the DRG	Mechanical allodynia	[105]
	Male Sprague–Dawley rats	HDAC4 retention in the cytoplasm	–	–	[53]
Spared nerve injury (SNI)	Male CD1 mice	HDAC1, BRD4 upregulation in the spinal cord	–	Mechanical allodynia, heat hyperalgesia	[54,57,58]
	Male CD1 mice	HDAC1 upregulation in the spinal microglia	–	Mechanical allodynia, heat hyperalgesia	[56]
	Male CD1 mice	HDAC1 upregulation in the spinal cord		Mechanical allodynia	[55]
	Male Sprague–Dawley rats	HDAC1–3 downregulation in the spinal cord	–	–	[4]
	Male and female C57BL/6J mice	H3K27ac ↓ in the lateral hypothalamus and nucleus accumbens	–	Mechanical allodynia, thermal hyperalgesia	[59]
	Male C57BL/6J mice	H3 hyperacetylation at the CXCL13 promoter	CXCL13 upregulation in DRG neurons	Mechanical allodynia, heat hyperalgesia	[95]
	Male C57BL/6 mice	HDAC overactivity	GAD65 downregulation in the spinal cord	Mechanical allodynia	[70]
Bone cancer pain	Female Wistar rats, female Sprague–Dawley rats	HDAC2 upregulation in spinal neurons and astroglia	Downregulation of KCC2 in the spinal cord	Mechanical allodynia	[82]
	Female Wistar or Sprague–Dawley rats	HDAC1 and HDAC2 upregulation in the DRG	MOP downregulation in the spinal cord	Mechanical allodynia, heat hyperalgesia	[85,86]
	Female Sprague–Dawley rats	HDAC1 and HDAC2 upregulation in the DRG	Glycogen synthase kinase 3β, TNF-α, IL-1β, IL-6 in DRG ↑	Mechanical allodynia	[47]
Cancer chemotherapy-induced neuropathic pain	Male Sprague–Dawley rats	Paclitaxel-induced, NF-κB-dependent H4 hyperacetylation at the promoter of the CX3CL1 gene	CX3CL1 upregulation in spinal neurons	Mechanical allodynia	[93]
	Male Sprague–Dawley rats, male C57BL/6 mice	pSTAT3- and p300-dependent H4 hyperacetylation at the promoter of the CXCL12 gene	CXCL12 upregulation in dorsal horn neurons	Mechanical allodynia	[94]
	Male C57BL/6J mice	Oxaliplatin-induced upregulation of the NRSF–NRSE–HDAC3 pathway	Downregulation of TREK1, TRAAK, K_v_1.1, and K_v_4.3 in DRG	Mechanical and cold allodynia	[80]
	Male Sprague–Dawley rats	Oxaliplatin-induced H4 hypoacetylation at the promoter of the TSC2 gene	Downregulation of TSC2 in the dorsal horn	Mechanical allodynia	[107]
Bortezomib-induced neuropathy	Male Sprague–Dawley rats, C57BL/6 mice	STAT3 ↑ → global acetylation of H3 and H4 ↑, hyperacetylation at the NLRP3 gene promoter	NLRP3 upregulation in the DRG	Mechanical allodynia	[99]
	Male Sprague–Dawley rats, C57BL/6 mice	SIRT1 downregulation → pSTAT3 → p-STAT3–p300 interaction → H3 and H4 hyperacetylation at the NALP1 gene promoter	NALP1 upregulation in the dorsal horn	Mechanical allodynia	[62]
	Male Sprague–Dawley rats	SIRT1 downregulation H4 hyperacetylation in the spinal cord	TNFα, IL-1β, IL-6 ↑, NF-κB phosphorylation	Mechanical allodynia	[65]
	Male Sprague–Dawley rats	H3 hypoacetylation at the AMPKa2 gene promoter	AMPKa2 downregulation in the dorsal horn	–	[106]
Stavudine-induced neuropathy	Male Wistar rats	Histone acetylation in H3K9 promoters ↓ in spinal cord	–	Mechanical allodynia, heat hyperalgesia	[50]
Diabetic neuropathy	Male Sprague–Dawley rats	SIRT1 downregulation → H3 hyperacetylation at the mGlu1/5 gene promoters	mGlu1/5 upregulation in spinal neurons	Mechanical allodynia, heat hyperalgesia	[48]
	Zucker diabetic (Type 2) fatty rats	Hperacetylation (H3K9) at the HMGB1 gene promoter	HMGB1 upregulation in the spinal cord and DRG	Mechanical allodynia, heat hyperalgesia	[96]
L5 ventral root transection	Male Sprague–Dawley rats, C57BL/6 mice	TNF-α–pSTAT3–p300 → H4 hyperacetylation at the Na_v_1.6 gene promoter	Na_v_1.6 upregulation in the DRG	Mechanical allodynia	[81]
Brachial plexus avulsion	Male Sprague–Dawley rats	HDAC activation, Akt-H3K9, Ac-H4K12 in DRG ↓	TNF-α, IL-1β, IL-6, TRPV1, TRPM8 in DRG ↑	Mechanical allodynia	[97]
Spinal cord injury	C57BL/6 mice	Histone hyperacetylation	TNF-α, IL-1β, IL-6 upregulation; IL-4, IL-10, IL-13 downregulation in the spinal cord	Mechanical allodynia	[98]
Trigeminal neuralgia	Female Sprague–Dawley rats	H3K9 acetylation in the trigeminal ganglion ↓	–	Mechanical allodynia	[67]
Trigeminal inflammatory compression	Male BALB/C mice	H3K9 acetylation in the trigeminal ganglion ↓	–	Whisker pad mechanical hypersensitivity	[66]
Chronic constriction injury (CCI) to the infraorbital nerve	Male Wistar rats	SIRT1 downregulation → H3K9ac ↑ at the promoter of the CaMKIIaα gene	CaMKIIaα downregulation in the central nucleus of the amygdala	–	[68]
	Male and female Sprague–Dawley rats	HDAC9 downregulation → H3K18ac ↑ at the promoter of the miR-203a-3p gene	miR-203a-3p → proprotein convertase 1 expression ↓ → β-endorphin downregulation in the hypothalamic arcuate nucleus	Mechanical allodynia	[87]

The upward and downward arrows mean increases and decreases of the mentioned factor or process, respectively. The horizontal arrows indicate consequences.

**Table 3:** Experimental models in which the effects of histone methylation were examined.

Neuropathy Model	Species	Epigenetic Alteration(s)	Epigenetically Regulated Change(s) in Gene Expression	Epigenetically Regulated Nociceptive Response(s)	Reference(s)
Chronic constriction injury (CCI)	Male C57BL/6J mice	MIF upregulation → G9a, SUV39H1 upregulation → H3K9me2/3 enrichment → increased CpG island methylation at the promoter of the tyrosine hydroxylase gene	Downregulation of tyrosine hydroxylase and dopamine in the ventral tegmental area	Mechanical allodynia, heat hyperalgesia	[119]
	Male C57BL/6J mice	CARM1 upregulation in DRG neurons	–	Mechanical allodynia, heat hyperalgesia	[109]
	Male Wistar rats	CGRP upregulation → EZH2 up-regulation, and H3K27me3 enrichment in microglia	Upregulation of several microglial activation markers	Mechanical allodynia, heat hyperalgesia	[115]
Spinal nerve ligation (SNL)	Male Sprague–Dawley rats	G9a upregulation → increased H3K9me2 occupancy at the promoters of K_v_1.4, K_v_4.2, K_v_7.2, and Ca^2+^-activated K^+^ channel genes	K_v_1.4, K_v_4.2, K_v_7.2, and Ca^2+^-activated K^+^ channel mRNA levels decrease in DRG neurons	Tactile allodynia, mechanical hyperalgesia	[45]
	Male C57BL/6J mice	G9a upregulation → increased H3K9me2 level	K_v_1.2 downregulation in DRG neurons	Mechanical allodynia, heat, and cold hyperalgesia	[110]
	Male Sprague–Dawley rats	Increased H3K9me2 level at the promoter of the MOP gene	MOP downregulation in the DRG	Mechanical allodynia	[113]
	C54BL/6J mice, Sprague–Dawley rats	Increase in G9a and H3K9me2 in the MOP, DOP, and KOP genes	MOP, DOP, KOP downregulation in the DRG	Mechanical allodynia, heat hyperalgesia	[112]
	Male C57BL/6J mice	CARM1 upregulation in DRG neurons	–	Mechanical allodynia, heat hyperalgesia	[109]
	Male and female Sprague–Dawley rats	CARM1 downregulation, H3R17me2 decrease at the promoter of the K_v_1.4 and K_v_4.2 genes	K_v_1.4 and K_v_4.2 downregulation in dorsal horn neurons	Mechanical allodynia	[111]
	Male Sprague–Dawley rats	KDM6B upregulation → H3K27me3 demethylation at the promoter of the IL-6 gene	IL-6 upregulation in the DRG and dorsal horn	Mechanical allodynia, heat hyperalgesia	[121]
	Male Sprague–Dawley rats	MLL1 upregulation and interaction with WDR5 → H3K4me3 enrichment at the promoter of the mGlu5 gene	mGlu5 upregulation in dorsal horn neurons	Mechanical allodynia	[125]
	Male Sprague–Dawley rats	Enrichment of H3K4me3, H3K9ac, reduction in H3K9me2, H3K27me3 at the promoter of the CB_2_ receptor gene	CB_2_ receptor upregulation in the DRG	–	[122]
Spared nerve injury (SNI)	Male and female C57BL/6J mice	H3K4me1 ↓ in the periaqueductal grey matter	–	Mechanical allodynia, thermal hyperalgesia	[59]
	Male C57BL/6J mice	G9a/GLP upregulation, increased DNA methylation in the tyrosine hydroxylase gene	Downregulation of tyrosine hydroxylase and dopamine in the ventral tegmental area	Mechanical allodynia	[118]
	Male Sprague–Dawley rats	MLL1 upregulation and interaction with WDR5 → H3K4me3 enrichment at the promoter of the mGlu5 gene	mGlu5 upregulation in dorsal horn neurons	Mechanical allodynia	[125]
Partial sciatic nerve ligation (PSNL)	Male Sprague–Dawley rats	EZH2 and H3K27me3 upregulation in dorsal horn neurons and microglia	–	Mechanical allodynia, heat hyperalgesia	[114]
	Male C57BL/6J mice	IL-6 → pSTAT3 and PU.1 upregulation → decreased enrichment of H3K27me3 at the promoter of the MCP-3/CCL7 gene	MCP-3/CCL7 upregulation in spinal astrocytes	Mechanical allodynia, heat hyperalgesia	[126]
Cancer chemotherapy-induced neuropathic pain	Male C57BL/6J mice	Cisplatin-induced decrease in H3.1K27me and H3K56me3 enrichment in microglia	–	Mechanical allodynia	[116]
	Male and female Sprague–Dawley rats	Paclitaxel-induced pNEK2 upregulation → KDM6 upregulation → demethylation of H3K27me3 at the promoter of the TRPV1 gene	TRPV1 upregulation in DRG neurons	Mechanical allodynia, heat hyperalgesia	[123]
	Male and female Sprague–Dawley rats	Paclitaxel-induced NAD^+^ phosphate oxidase 4 → PRMT5 upregulation → H3R2me2 enrichment then WDR5-mediated H3K4me3 enrichment at the TRPV1 gene promoter	TRPV1 upregulation in DRG neurons	Mechanical allodynia, heat hyperalgesia	[124]
Bortezomib-induced neuropathy	Male C57BL/6J mice	Prokineticins → KDM6A upregulation in the spinal cord	–	Mechanical allodynia	[117]

The upward and downward arrows mean increases and decreases of the factor or process, respectively. The horizontal arrows indicate consequences.

**Table 4:** Summary of epigenetic enzyme inhibitors tested in models of neuropathic pain.

Enzyme	Inhibitor	Model(s)	Reference(s)
DNA methyltransferase (DNMT)	5-Azacytidine	CCI/SNL/Chronic low back pain	[9,13,41]
	5-Aza-deoxycytidine	CCI	[27,28]
	Decitabine	Bone cancer	[8]
	RG108	CCI/SNL	[15,30]
Histone deacetylase (HDAC)	MS275	SNL/PSNL/Stavudine-induced neuropathy/Trigeminal inflammatory compression/SNI	[50,66,70]
	MGCD0103	SNL, PSNL, Stavudine-induced neuropathy	[50]
	Baicalin	SNL	[51]
	Sodium butyrate	CCI	[52]
	LG325	SNI	[54]
	Zingiberene	SNI	[56]
	SAHA	SNI/Trigeminal inflammatory compression/SNL/PSNL/CCI/Bone cancer	[28,45,47,57,66,70,71,76,79,86]
	Carbamazepine	Trigeminal neuralgia	[67]
	CAY10683	SNL	[72]
	Trichostatin A	PSNL/Bone cancer/CCI	[76,82,83,84,85]
	Valproic acid	PSNL	[76,84]
	JNJ26481585 (quinostat)	Chemotrerapy-induced neuropathy	[129]
Bromodomain and extraterminal (BET)	i-BET762	SNI	[57]
	JQ1	Spinal cord injury	[98]
Histone deacetylase-bromodomain-containing proteins (HDAC/BRD)	SUM52	SNI	[58]
	SUM35	SNI	[58]
Silent information regulator (SIRT)	Resveratrol (activator)	Bortezomib-induced neuropathy	[62]
	SRT1720 (activator)	Bortezomib-induced neuropathy/CCI	[62,64]
Histone acetyltransferase (HAT)	Anacardic acid	PSNL	[88,89]
Histone methyltranserase G9a	UNC0638	SNL	[45,113]
	BIX01294	SNL	[112]
Enhancer of the histone methyltransferase zeste homolog-2 (EZH2)	GSK503	SNL	[45]
Lysine demethylase 7A (KDM7A)	NCDM-64	Chemotherapy-induced neuropathy	[116]

## Abbreviations

5mC	5-methylcytosine
5hmC	5-hydroxymethylcytosine
AMP	adenosine monophosphate
AMPK	AMP-activated kinase
BDNF	brain-derived neurotrophic factor
BET	bromodomain and extraterminal
BRD	bromodomain-containing proteins
CaMKIIaα	Ca^2+^/calmodulin-dependent protein kinase IIaα
CARM	coactivator-associated arginine methyltransferase
CBP	CREB binding protein
CCI	chronic constriction injury
CCL	C-C motif chemokine ligand
CCR	C-C motif chemokine receptor
Cdk5	cyclin-dependent kinase 5
CXCR	chemokine CC motif receptor
CGRP	calcitonin gene-related peptide
COX-2	cyclooxygenase 2
CREB	cyclic AMP-response element binding protein
CX3CL	chemokine C-X3-C motif ligand
CXCL	C-X-C motif ligand
DDR1	discoidin domain receptor type 1
DNA	deoxyribonucleic acid
DNMT	DNA methyltransferase
DOP	delta opioid receptor
DRG	dorsal root ganglia
ERK	extracellular signal-regulated kinase
ET_B_	endothelin type B
ETS	erythroblast transformation specific
ETSI	erythroblast transformation specific proto-oncogene I
EZH2	enhancer of the histone methyltransferase zeste homolog-2
GABA	gamma-aminobutyric acid
GAD65	glutamic acid decarboxylase 65
GAD67	glutamic acid decarboxylase 67
GFAP	glial fibrillary acidic protein
GLP	G9a-like protein
GLT-1	glutamate transporter type 1
GNAT	general control non-derepressible 5-related acetyltransferases
H3K	histone H3 at lysine
H3K9	histone H3 at lysine 9
H3K9me2	dimethylation of histone H3 at lysine 9
H3K27me3	trimethylation of histone H3 at lysine 27
H4K	histone H4 at lysine
HAT	histone acetyltransferase
HDAC	histone deacetylase
HDM	histone demethylase
HMGB	high-mobility group box
HMT	histone methyltransferase
HOXA6	homeobox A6
IL	interleukin
iNOS	inducible nitric oxide synthase
JMJD	jumonji domain-containing protein
JNK1	C-Jun N-terminal kinase 1
K_2P_	two-pore-domain K^+^ channel
KCC2	K^+^–Cl^−^-cotransporter 2
KDM	lysine demethylase enzyme
KOP	kappa opioid receptor
MBD	methyl-CpG-binding domain
MCP	monocyte chemotactic protein
MeCP2	methyl-CpG-binding protein 2
mGlu	metabotropic glutamate receptor
MIF	macrophage migration inhibiting factor
MIP	macrophage inflammatory protein
MLL1	mixed lineage leukemia type 1
MOP	mu opioid receptor
mTOR	molecular target of rapamycin
NAD^+^	nicotinamide adenine dinucleotide
NALP	NACHT leucine-rich-repeat protein
NFATc2	nuclear factor of activated T cells type 2
NF-κB	nuclear factor kappa B
NGF	nerve growth factor
NEK	NIMA-related kinase
NLRP3	nucleotide oligomerization domain-like receptor protein 3
NMDA	N-methyl-D-aspartate
NOD	nucleotide oligomerization domain
NRSF	neuron-restrictive silencer factor
NRSE	neuron-restrictive silencer element
OCT1	octamer transcription factor 1
PADI	peptidyl arginine deiminase
pERK1/2	phosphorylated extracellular signal-regulated kinase 1 and 2
PGC-1α	peroxisome proliferator-activated receptor-γ coactivator-1α
PKA	protein kinase A
PKC	protein kinase C
PPAR	peroxisome proliferator-activated receptor
pRSK2	phosphorylated p90 ribosomal S6 kinase 2
PRMT	protein arginine methyltransferase
PSNL	partial sciatic nerve ligation
pSTAT3	phosphorylated transducer and activator of transcription-3
RE	response element
REST	repressor element 1-silencing transcription factor
RNA	ribonucleic acid
mRNA	messenger ribonucleic acid
SAHA	suberoylanilide hydroxamic acid
SETDB1	SET domain bifurcated histone lysine methyltransferase 1
SGK1	serum- and glucocorticoid-inducible kinase 1
shRNA	short hairpin ribonucleic acid
siRNA	small interfering ribonucleic acid
SIRT	silent information regulator
SNI	spared nerve injury
SNL	spinal nerve ligation
Sp1	specificity protein 1
SPARC	secreted protein acidic rich in cysteine
SOX10	SRY-related HMG-box 10
STAT	signal transducer and activator
SUV39H1	suppressor of variegation 3-9 homolog 1
TET	ten-eleven translocation
TLR4	toll-like receptor 4
TNF-α	tumor necrosis factor α
TRP	transient receptor potential
TRPA1	transient receptor potential ankyrin 1
TRPM8	transient receptor potential melastatin 8
TRPV1	transient receptor potential vanilloid 1
TSC2	tuberous sclerosis complex protein 2
TXNIP	thioredoxin-interacting protein
VEGF	vascular endothelial growth factor
VEGFR	vascular endothelial growth factor receptor
WDR5	WD repeat-containing protein 5
ZEB1	zinc-finger E-box-binding homeobox 1
ZNF	zinc-finger protein

## References

[1] Biswas S., Rao C.M. (2018). Epigenetic Tools (The Writers, The Readers and The Erasers) and Their Implications in Cancer Therapy. Eur. J. Pharmacol..

[2] Bayraktar G., Kreutz M.R. (2018). Neuronal DNA Methyltransferases: Epigenetic Mediators between Synaptic Activity and Gene Expression?. Neuroscientist.

[3] Tahiliani M., Koh K.P., Shen Y., Pastor W.A., Bandukwala H., Brudno Y., Agarwal S., Iyer L.M., Liu D.R., Aravind L. (2009). Conversion of 5-Methylcytosine to 5-Hydroxymethylcytosine in Mammalian DNA by MLL Partner TET1. Science.

[4] Tochiki K.K., Cunningham J., Hunt S.P., Géranton S.M. (2012). The Expression of Spinal Methyl-CpG-Binding Protein 2, DNA Methyltransferases and Histone Deacetylases Is Modulated in Persistent Pain States. Mol. Pain.

[5] Sun L., Zhao J.-Y., Gu X., Liang L., Wu S., Mo K., Feng J., Guo W., Zhang J., Bekker A. (2017). Nerve Injury–Induced Epigenetic Silencing of Opioid Receptors Controlled by DNMT3a in Primary Afferent Neurons. Pain.

[6] Zhao J.-Y., Liang L., Gu X., Li Z., Wu S., Sun L., Atianjoh F.E., Feng J., Mo K., Jia S. (2017). DNA Methyltransferase DNMT3a Contributes to Neuropathic Pain by Repressing Kcna2 in Primary Afferent Neurons. Nat. Commun..

[7] Sun L., Gu X., Pan Z., Guo X., Liu J., Atianjoh F.E., Wu S., Mo K., Xu B., Liang L. (2019). Contribution of DNMT1 to Neuropathic Pain Genesis Partially through Epigenetically Repressing *Kcna2* in Primary Afferent Neurons. J. Neurosci..

[8] Miao X.-R., Fan L.-C., Wu S., Mao Q.-X., Li Z., Lutz B.M., Xu J., Lu Z.-J., Tao Y.-X. (2017). DNMT3a Contributes to the Development and Maintenance of Bone Cancer Pain by Silencing Kv1.2 Expression in Spinal Cord Dorsal Horn. Mol. Pain.

[9] Wang Y., Liu C., Guo Q.-L., Yan J.-Q., Zhu X.-Y., Huang C.-S., Zou W.-Y. (2011). Intrathecal 5-Azacytidine Inhibits Global DNA Methylation and Methyl- CpG-Binding Protein 2 Expression and Alleviates Neuropathic Pain in Rats Following Chronic Constriction Injury. Brain Res..

[10] Mo K., Wu S., Gu X., Xiong M., Cai W., Atianjoh F.E., Jobe E.E., Zhao X., Tu W.-F., Tao Y.-X. (2018). MBD1 Contributes to the Genesis of Acute Pain and Neuropathic Pain by Epigenetic Silencing of *Oprm1* and *Kcna2* Genes in Primary Sensory Neurons. J. Neurosci..

[11] Chamessian A.G., Qadri Y.J., Cummins M., Hendrickson M., Berta T., Buchheit T., Van De Ven T. (2017). 5-Hydroxymethylcytosine (5hmC) and Ten-Eleven Translocation 1–3 (TET1–3) Proteins in the Dorsal Root Ganglia of Mouse: Expression and Dynamic Regulation in Neuropathic Pain. Somatosens. Mot. Res..

[12] Hsieh M.-C., Lai C.-Y., Ho Y.-C., Wang H.-H., Cheng J.-K., Chau Y.-P., Peng H.-Y. (2016). Tet1-Dependent Epigenetic Modification of BDNF Expression in Dorsal Horn Neurons Mediates Neuropathic Pain in Rats. Sci. Rep..

[13] Li X., Liu D., Dai Z., You Y., Chen Y., Lei C., Lv Y., Wang Y. (2023). Intraperitoneal 5-Azacytidine Alleviates Nerve Injury-Induced Pain in Rats by Modulating DNA Methylation. Mol. Neurobiol..

[14] Gölzenleuchter M., Kanwar R., Zaibak M., Al Saiegh F., Hartung T., Klukas J., Smalley R.L., Cunningham J.M., Figueroa M.E., Schroth G.P. (2015). Plasticity of DNA Methylation in a Nerve Injury Model of Pain. Epigenetics.

[15] Garriga J., Laumet G., Chen S.-R., Zhang Y., Madzo J., Issa J.-P.J., Pan H.-L., Jelinek J. (2018). Nerve Injury-Induced Chronic Pain Is Associated with Persistent DNA Methylation Reprogramming in Dorsal Root Ganglion. J. Neurosci..

[16] Manners M.T., Ertel A., Tian Y., Ajit S.K. (2016). Genome-Wide Redistribution of MeCP2 in Dorsal Root Ganglia after Peripheral Nerve Injury. Epigenetics Chromatin.

[17] Tajerian M., Alvarado S., Millecamps M., Vachon P., Crosby C., Bushnell M.C., Szyf M., Stone L.S. (2013). Peripheral Nerve Injury Is Associated with Chronic, Reversible Changes in Global DNA Methylation in the Mouse Prefrontal Cortex. PLoS ONE.

[18] Massart R., Dymov S., Millecamps M., Suderman M., Gregoire S., Koenigs K., Alvarado S., Tajerian M., Stone L.S., Szyf M. (2016). Overlapping Signatures of Chronic Pain in the DNA Methylation Landscape of Prefrontal Cortex and Peripheral T Cells. Sci. Rep..

[19] Grégoire S., Millecamps M., Naso L., Do Carmo S., Cuello A.C., Szyf M., Stone L.S. (2017). Therapeutic Benefits of the Methyl Donor S-Adenosylmethionine on Nerve Injury–Induced Mechanical Hypersensitivity and Cognitive Impairment in Mice. Pain.

[20] Topham L., Gregoire S., Kang H., Salmon-Divon M., Lax E., Millecamps M., Szyf M., Stone L.S. (2020). The Transition from Acute to Chronic Pain: Dynamic Epigenetic Reprogramming of the Mouse Prefrontal Cortex up to 1 Year after Nerve Injury. Pain.

[21] Liu R., Wu X., He X., Wang R., Yin X., Zhou F., Ji M., Shen J. (2021). Contribution of DNA Methyltransferases to Spared Nerve Injury Induced Depression Partially through Epigenetically Repressing Bdnf in Hippocampus: Reversal by Ketamine. Pharmacol. Biochem. Behav..

[22] Rodrigues D., Monteiro C., Cardoso-Cruz H., Galhardo V. (2023). Altered Brain Expression of DNA Methylation and Hydroxymethylation Epigenetic Enzymes in a Rat Model of Neuropathic Pain. Int. J. Mol. Sci..

[23] Jang J.-H., Song E.-M., Do Y.-H., Ahn S., Oh J.-Y., Hwang T.-Y., Ryu Y., Jeon S., Song M.-Y., Park H.-J. (2021). Acupuncture Alleviates Chronic Pain and Comorbid Conditions in a Mouse Model of Neuropathic Pain: The Involvement of DNA Methylation in the Prefrontal Cortex. Pain.

[24] Ping X., Xie J., Yuan C., Jin X. (2023). Electroacupuncture Induces Bilateral S1 and ACC Epigenetic Regulation of Genes in a Mouse Model of Neuropathic Pain. Biomedicines.

[25] Chen W., Lan T., Sun Q., Zhang Y., Shen D., Hu T., Liu J., Chong Y., Wang P., Li Q. (2021). Whole Genomic DNA Methylation Profiling of CpG Sites in Promoter Regions of Dorsal Root Ganglion in Diabetic Neuropathic Pain Mice. J. Mol. Neurosci..

[26] Stenz L., Carré J.L., Luthi F., Vuistiner P., Burrus C., Paoloni-Giacobino A., Léger B. (2022). Genome-Wide Epigenomic Analyses in Patients With Nociceptive and Neuropathic Chronic Pain Subtypes Reveals Alterations in Methylation of Genes Involved in the Neuro-Musculoskeletal System. J. Pain.

[27] Zhou X.-L., Yu L.-N., Wang Y., Tang L.-H., Peng Y.-N., Cao J.-L., Yan M. (2014). Increased Methylation of the MOR Gene Proximal Promoter in Primary Sensory Neurons Plays a Crucial Role in the Decreased Analgesic Effect of Opioids in Neuropathic Pain. Mol. Pain.

[28] Sun N., Yu L., Gao Y., Ma L., Ren J., Liu Y., Gao D.S., Xie C., Wu Y., Wang L. (2021). MeCP2 Epigenetic Silencing of Oprm1 Gene in Primary Sensory Neurons under Neuropathic Pain Conditions. Front. Neurosci..

[29] Yuan J., Wen J., Wu S., Mao Y., Mo K., Li Z., Su S., Gu H., Ai Y., Bekker A. (2019). Contribution of Dorsal Root Ganglion Octamer Transcription Factor 1 to Neuropathic Pain after Peripheral Nerve Injury. Pain.

[30] Shao C., Gao Y., Jin D., Xu X., Tan S., Yu H., Zhao Q., Zhao L., Wang W., Wang D. (2017). DNMT3a Methylation in Neuropathic Pain. J. Pain Res..

[31] Wu Q., Wei G., Ji F., Jia S., Wu S., Guo X., He L., Pan Z., Miao X., Mao Q. (2019). TET1 Overexpression Mitigates Neuropathic Pain Through Rescuing the Expression of μ-Opioid Receptor and Kv1.2 in the Primary Sensory Neurons. Neurotherapeutics.

[32] Mao Q., Wu S., Gu X., Du S., Mo K., Sun L., Cao J., Bekker A., Chen L., Tao Y. (2019). DNMT3a-triggered Downregulation of K_2p_ 1.1 Gene in Primary Sensory Neurons Contributes to Paclitaxel-induced Neuropathic Pain. Int. J. Cancer.

[33] Manners M.T., Tian Y., Zhou Z., Ajit S.K. (2015). MicroRNAs Downregulated in Neuropathic Pain Regulate MeCP2 and BDNF Related to Pain Sensitivity. FEBS Open Bio.

[34] Wang Y., Lin Z.-P., Zheng H.-Z., Zhang S., Zhang Z.-L., Chen Y., You Y.-S., Yang M.-H. (2016). Abnormal DNA Methylation in the Lumbar Spinal Cord Following Chronic Constriction Injury in Rats. Neurosci. Lett..

[35] Hsieh M.-C., Ho Y.-C., Lai C.-Y., Chou D., Wang H.-H., Chen G.-D., Lin T.-B., Peng H.-Y. (2017). Melatonin Impedes Tet1-Dependent mGluR5 Promoter Demethylation to Relieve Pain. J. Pineal Res..

[36] Zhang H.-H., Hu J., Zhou Y.-L., Qin X., Song Z.-Y., Yang P.-P., Hu S., Jiang X., Xu G.-Y. (2015). Promoted Interaction of Nuclear Factor-κB With Demethylated Purinergic P2X3 Receptor Gene Contributes to Neuropathic Pain in Rats With Diabetes. Diabetes.

[37] Chen W., Wang X., Sun Q., Zhang Y., Liu J., Hu T., Wu W., Wei C., Liu M., Ding Y. (2022). The Upregulation of NLRP3 Inflammasome in Dorsal Root Ganglion by Ten-Eleven Translocation Methylcytosine Dioxygenase 2 (TET2) Contributed to Diabetic Neuropathic Pain in Mice. J. Neuroinflammation.

[38] Viet C.T., Ye Y., Dang D., Lam D.K., Achdjian S., Zhang J., Schmidt B.L. (2011). Re-Expression of the Methylated EDNRB Gene in Oral Squamous Cell Carcinoma Attenuates Cancer-Induced Pain. Pain.

[39] Deng J., Ding H., Long J., Lin S., Liu M., Zhang X., Xin W., Ruan X. (2020). Oxaliplatin-induced Neuropathic Pain Involves HOXA6 via a TET1 -dependent Demethylation of the SOX10 Promoter. Int. J. Cancer.

[40] Chen Y.-Y., Jiang K.-S., Bai X.-H., Liu M., Lin S.-Y., Xu T., Wei J.-Y., Li D., Xiong Y.-C., Xin W.-J. (2021). ZEB1 Induces Ddr1 Promoter Hypermethylation and Contributes to the Chronic Pain in Spinal Cord in Rats Following Oxaliplatin Treatment. Neurochem. Res..

[41] Tajerian M., Alvarado S., Millecamps M., Dashwood T., Anderson K.M., Haglund L., Ouellet J., Szyf M., Stone L.S. (2011). DNA Methylation of SPARC and Chronic Low Back Pain. Mol. Pain.

[42] Sukenaga N., Ikeda-Miyagawa Y., Tanada D., Tunetoh T., Nakano S., Inui T., Satoh K., Okutani H., Noguchi K., Hirose M. (2016). Correlation Between DNA Methylation of TRPA1 and Chronic Pain States in Human Whole Blood Cells. Pain Med..

[43] Khangura R.K., Bali A., Jaggi A.S., Singh N. (2017). Histone Acetylation and Histone Deacetylation in Neuropathic Pain: An Unresolved Puzzle?. Eur. J. Pharmacol..

[44] Torres-Perez J.V., Irfan J., Febrianto M.R., Di Giovanni S., Nagy I. (2021). Histone Post-Translational Modifications as Potential Therapeutic Targets for Pain Management. Trends Pharmacol. Sci..

[45] Laumet G., Garriga J., Chen S.-R., Zhang Y., Li D.-P., Smith T.M., Dong Y., Jelinek J., Cesaroni M., Issa J.-P. (2015). G9a Is Essential for Epigenetic Silencing of K+ Channel Genes in Acute-to-Chronic Pain Transition. Nat. Neurosci..

[46] Maiarù M., Morgan O.B., Tochiki K.K., Hobbiger E.J., Rajani K., Overington D.W.U., Géranton S.M. (2016). Complex Regulation of the Regulator of Synaptic Plasticity Histone Deacetylase 2 in the Rodent Dorsal Horn after Peripheral Injury. J. Neurochem..

[47] He X.-T., Hu X.-F., Zhu C., Zhou K.-X., Zhao W.-J., Zhang C., Han X., Wu C.-L., Wei Y.-Y., Wang W. (2020). Suppression of Histone Deacetylases by SAHA Relieves Bone Cancer Pain in Rats via Inhibiting Activation of Glial Cells in Spinal Dorsal Horn and Dorsal Root Ganglia. J. Neuroinflammation.

[48] Zhou C.-H., Zhang M.-X., Zhou S.-S., Li H., Gao J., Du L., Yin X.-X. (2017). SIRT1 Attenuates Neuropathic Pain by Epigenetic Regulation of mGluR1/5 Expressions in Type 2 Diabetic Rats. Pain.

[49] Kami K., Taguchi S., Tajima F., Senba E. (2016). Histone Acetylation in Microglia Contributes to Exercise-Induced Hypoalgesia in Neuropathic Pain Model Mice. J. Pain.

[50] Denk F., Huang W., Sidders B., Bithell A., Crow M., Grist J., Sharma S., Ziemek D., Rice A.S.C., Buckley N.J. (2013). HDAC Inhibitors Attenuate the Development of Hypersensitivity in Models of Neuropathic Pain. Pain.

[51] Cherng C.-H., Lee K.-C., Chien C.-C., Chou K.-Y., Cheng Y.-C., Hsin S.-T., Lee S.-O., Shen C.-H., Tsai R.-Y., Wong C.-S. (2014). Baicalin Ameliorates Neuropathic Pain by Suppressing HDAC1 Expression in the Spinal Cord of Spinal Nerve Ligation Rats. J. Formos. Med. Assoc..

[52] Kukkar A., Singh N., Jaggi A.S. (2014). Attenuation of Neuropathic Pain by Sodium Butyrate in an Experimental Model of Chronic Constriction Injury in Rats. J. Formos. Med. Assoc..

[53] Lin T.-B., Hsieh M.-C., Lai C.-Y., Cheng J.-K., Chau Y.-P., Ruan T., Chen G.-D., Peng H.-Y. (2015). Modulation of Nerve Injury–Induced HDAC4 Cytoplasmic Retention Contributes to Neuropathic Pain in Rats. Anesthesiology.

[54] Sanna M.D., Guandalini L., Romanelli M.N., Galeotti N. (2017). The New HDAC1 Inhibitor LG325 Ameliorates Neuropathic Pain in a Mouse Model. Pharmacol. Biochem. Behav..

[55] Sanna M.D., Galeotti N. (2018). The HDAC1/c-JUN Complex Is Essential in the Promotion of Nerve Injury-Induced Neuropathic Pain through JNK Signaling. Eur. J. Pharmacol..

[56] Borgonetti V., Governa P., Manetti F., Galeotti N. (2023). Zingiberene, a Non-Zinc-Binding Class I HDAC Inhibitor: A Novel Strategy for the Management of Neuropathic Pain. Phytomedicine.

[57] Borgonetti V., Galeotti N. (2021). Combined Inhibition of Histone Deacetylases and BET Family Proteins as Epigenetic Therapy for Nerve Injury-Induced Neuropathic Pain. Pharmacol. Res..

[58] Borgonetti V., Meacci E., Pierucci F., Romanelli M.N., Galeotti N. (2022). Dual HDAC/BRD4 Inhibitors Relieves Neuropathic Pain by Attenuating Inflammatory Response in Microglia After Spared Nerve Injury. Neurotherapeutics.

[59] Bryant S., Balouek J., Geiger L.T., Barker D.J., Peña C.J. (2023). Neuropathic Pain as a Trigger for Histone Modifications in Limbic Circuitry. Genes Brain Behav..

[60] Yin Q., Lu F.-F., Zhao Y., Cheng M.-Y., Fan Q., Cui J., Liu L., Cheng W., Yan C.-D. (2013). Resveratrol Facilitates Pain Attenuation in a Rat Model of Neuropathic Pain Through the Activation of Spinal Sirt1: *Reg*. Anesth. Pain Med..

[61] Shao H., Xue Q., Zhang F., Luo Y., Zhu H., Zhang X., Zhang H., Ding W., Yu B. (2014). Spinal SIRT1 Activation Attenuates Neuropathic Pain in Mice. PLoS ONE.

[62] Chen K., Fan J., Luo Z.-F., Yang Y., Xin W.-J., Liu C.-C. (2018). Reduction of SIRT1 Epigenetically Upregulates NALP1 Expression and Contributes to Neuropathic Pain Induced by Chemotherapeutic Drug Bortezomib. J. Neuroinflammation.

[63] Lv C., Hu H.-Y., Zhao L., Zheng H., Luo X.-Z., Zhang J. (2015). Intrathecal SRT1720, a SIRT1 Agonist, Exerts Anti-Hyperalgesic and Anti-Inflammatory Effects on Chronic Constriction Injury-Induced Neuropathic Pain in Rats. Int. J. Clin. Exp. Med..

[64] Zheng H.-L., Sun S.-Y., Jin T., Zhang M., Zeng Y., Liu Q., Yang K., Wei R., Pan Z., Lin F. (2023). Transcription Factor ETS Proto-Oncogene 1 Contributes to Neuropathic Pain by Regulating Histone Deacetylase 1 in Primary Afferent Neurons. Mol. Pain.

[65] Gui Y., Zhang J., Chen L., Duan S., Tang J., Xu W., Li A. (2018). Icariin, a Flavonoid with Anti-Cancer Effects, Alleviated Paclitaxel-Induced Neuropathic Pain in a SIRT1-Dependent Manner. Mol. Pain.

[66] Danaher R.J., Zhang L., Donley C.J., Laungani N.A., Hui S.E., Miller C.S., Westlund K.N. (2018). Histone Deacetylase Inhibitors Prevent Persistent Hypersensitivity in an Orofacial Neuropathic Pain Model. Mol. Pain.

[67] Wei W., Liu Y., Qiu Y., Chen M., Wang Y., Han Z., Chai Y. (2022). Characterization of Acetylation of Histone H3 at Lysine 9 in the Trigeminal Ganglion of a Rat Trigeminal Neuralgia Model. Oxid. Med. Cell. Longev..

[68] Zhou C., Wu Y., Ding X., Shi N., Cai Y., Pan Z.Z. (2020). SIRT1 Decreases Emotional Pain Vulnerability with Associated CaMKIIα Deacetylation in Central Amygdala. J. Neurosci..

[69] Zhang Z., Cai Y.-Q., Zou F., Bie B., Pan Z.Z. (2011). Epigenetic Suppression of GAD65 Expression Mediates Persistent Pain. Nat. Med..

[70] Shen X., Liu Y., Xu S., Zhao Q., Wu H., Guo X., Shen R., Wang F. (2014). Menin Regulates Spinal Glutamate-GABA Balance through GAD65 Contributing to Neuropathic Pain. Pharmacol. Rep..

[71] Cui S.-S., Lu R., Zhang H., Wang W., Ke J.-J. (2016). Suberoylanilide Hydroxamic Acid Prevents Downregulation of Spinal Glutamate Transporter-1 and Attenuates Spinal Nerve Ligation-Induced Neuropathic Pain Behavior. NeuroReport.

[72] Lu R., Cui S.-S., Wang X.-X., Chen L., Liu F., Gao J., Wang W. (2021). Astrocytic C-Jun N-Terminal Kinase-Histone Deacetylase-2 Cascade Contributes to Glutamate Transporter-1 Decrease and Mechanical Allodynia Following Peripheral Nerve Injury in Rats. Brain Res. Bull..

[73] Willis D.E., Wang M., Brown E., Fones L., Cave J.W. (2016). Selective Repression of Gene Expression in Neuropathic Pain by the Neuron-Restrictive Silencing Factor/Repressor Element-1 Silencing Transcription (NRSF/REST). Neurosci. Lett..

[74] Uchida H., Sasaki K., Ma L., Ueda H. (2010). Neuron-Restrictive Silencer Factor Causes Epigenetic Silencing of Kv4.3 Gene after Peripheral Nerve Injury. Neuroscience.

[75] Uchida H., Ma L., Ueda H. (2010). Epigenetic Gene Silencing Underlies C-Fiber Dysfunctions in Neuropathic Pain. J. Neurosci..

[76] Matsushita Y., Araki K., Omotuyi O.I., Mukae T., Ueda H. (2013). HDAC Inhibitors Restore C-Fibre Sensitivity in Experimental Neuropathic Pain Model: Epigenetic Repression in Neuropathic Pain. Br. J. Pharmacol..

[77] Mucha M., Ooi L., Linley J.E., Mordaka P., Dalle C., Robertson B., Gamper N., Wood I.C. (2010). Transcriptional Control of *KCNQ* Channel Genes and the Regulation of Neuronal Excitability. J. Neurosci..

[78] Rose K., Ooi L., Dalle C., Robertson B., Wood I.C., Gamper N. (2011). Transcriptional Repression of the M Channel Subunit Kv7.2 in Chronic Nerve Injury. Pain.

[79] Li Z., Guo Y., Ren X., Rong L., Huang M., Cao J., Zang W. (2019). HDAC2, but Not HDAC1, Regulates Kv1.2 Expression to Mediate Neuropathic Pain in CCI Rats. Neuroscience.

[80] Pereira V., Lamoine S., Cuménal M., Lolignier S., Aissouni Y., Pizzoccaro A., Prival L., Balayssac D., Eschalier A., Bourinet E. (2021). Epigenetics Involvement in Oxaliplatin-Induced Potassium Channel Transcriptional Downregulation and Hypersensitivity. Mol. Neurobiol..

[81] Ding H.-H., Zhang S.-B., Lv Y.-Y., Ma C., Liu M., Zhang K.-B., Ruan X.-C., Wei J.-Y., Xin W.-J., Wu S.-L. (2019). TNF-α/STAT3 Pathway Epigenetically Upregulates Nav1.6 Expression in DRG and Contributes to Neuropathic Pain Induced by L5-VRT. J. Neuroinflammation.

[82] Hou X., Weng Y., Wang T., Ouyang B., Li Y., Song Z., Pan Y., Zhang Z., Zou W., Huang C. (2018). Suppression of HDAC2 in Spinal Cord Alleviates Mechanical Hyperalgesia and Restores KCC2 Expression in a Rat Model of Bone Cancer Pain. Neuroscience.

[83] Ouyang B., Chen D., Hou X., Wang T., Wang J., Zou W., Song Z., Huang C., Guo Q., Weng Y. (2019). Normalizing HDAC2 Levels in the Spinal Cord Alleviates Thermal and Mechanical Hyperalgesia After Peripheral Nerve Injury and Promotes GAD65 and KCC2 Expression. Front. Neurosci..

[84] Uchida H., Matsushita Y., Araki K., Mukae T., Ueda H. (2015). Histone Deacetylase Inhibitors Relieve Morphine Resistance in Neuropathic Pain after Peripheral Nerve Injury. J. Pharmacol. Sci..

[85] Hou X., Weng Y., Ouyang B., Ding Z., Song Z., Zou W., Huang C., Guo Q. (2017). HDAC Inhibitor TSA Ameliorates Mechanical Hypersensitivity and Potentiates Analgesic Effect of Morphine in a Rat Model of Bone Cancer Pain by Restoring μ-Opioid Receptor in Spinal Cord. Brain Res..

[86] He X.-T., Zhou K.-X., Zhao W.-J., Zhang C., Deng J.-P., Chen F.-M., Gu Z.-X., Li Y.-Q., Dong Y.-L. (2018). Inhibition of Histone Deacetylases Attenuates Morphine Tolerance and Restores MOR Expression in the DRG of BCP Rats. Front. Pharmacol..

[87] Tao Y., Zhang Y., Jin X., Hua N., Liu H., Qi R., Huang Z., Sun Y., Jiang D., Snutch T.P. (2023). Epigenetic Regulation of Beta-Endorphin Synthesis in Hypothalamic Arcuate Nucleus Neurons Modulates Neuropathic Pain in a Rodent Pain Model. Nat. Commun..

[88] Kiguchi N., Kobayashi Y., Maeda T., Fukazawa Y., Tohya K., Kimura M., Kishioka S. (2012). Epigenetic Augmentation of the Macrophage Inflammatory Protein 2/C-X-C Chemokine Receptor Type 2 Axis through Histone H3 Acetylation in Injured Peripheral Nerves Elicits Neuropathic Pain. J. Pharmacol. Exp. Ther..

[89] Kiguchi N., Kobayashi Y., Saika F., Kishioka S. (2013). Epigenetic Upregulation of CCL2 and CCL3 via Histone Modifications in Infiltrating Macrophages after Peripheral Nerve Injury. Cytokine.

[90] Kiguchi N., Kobayashi Y., Kadowaki Y., Fukazawa Y., Saika F., Kishioka S. (2014). Vascular Endothelial Growth Factor Signaling in Injured Nerves Underlies Peripheral Sensitization in Neuropathic Pain. J. Neurochem..

[91] Uchida H., Matsushita Y., Ueda H. (2013). Epigenetic Regulation of BDNF Expression in the Primary Sensory Neurons after Peripheral Nerve Injury: Implications in the Development of Neuropathic Pain. Neuroscience.

[92] Tao W., Zhou W., Wang Y., Sun T., Wang H., Zhang Z., Jin Y. (2016). Histone Deacetylase Inhibitor-Induced Emergence of Synaptic δ-Opioid Receptors and Behavioral Antinociception in Persistent Neuropathic Pain. Neuroscience.

[93] Li D., Huang Z.-Z., Ling Y.-Z., Wei J.-Y., Cui Y., Zhang X.-Z., Zhu H.-Q., Xin W.-J. (2015). Up-Regulation of CX3CL1 via Nuclear Factor-κB–Dependent Histone Acetylation Is Involved in Paclitaxel-Induced Peripheral Neuropathy. Anesthesiology.

[94] Xu T., Zhang X.-L., Ou-Yang H.-D., Li Z.-Y., Liu C.-C., Huang Z.-Z., Xu J., Wei J.-Y., Nie B.-L., Ma C. (2017). Epigenetic Upregulation of CXCL12 Expression Mediates Antitubulin Chemotherapeutics–Induced Neuropathic Pain. Pain.

[95] Ma L., Yu L., Jiang B.-C., Wang J., Guo X., Huang Y., Ren J., Sun N., Gao D.S., Ding H. (2021). ZNF382 Controls Mouse Neuropathic Pain via Silencer-Based Epigenetic Inhibition of *Cxcl13* in DRG Neurons. J. Exp. Med..

[96] Thakur V., Sadanandan J., Chattopadhyay M. (2020). High-Mobility Group Box 1 Protein Signaling in Painful Diabetic Neuropathy. Int. J. Mol. Sci..

[97] Zhao Y., Wu T. (2018). Histone Deacetylase Inhibition Inhibits Brachial Plexus Avulsion-Induced Neuropathic Pain: HDAC, Akt, & Neuropathic Pain. Muscle Nerve.

[98] Sánchez-Ventura J., Amo-Aparicio J., Navarro X., Penas C. (2019). BET Protein Inhibition Regulates Cytokine Production and Promotes Neuroprotection after Spinal Cord Injury. J. Neuroinflammation.

[99] Liu C.-C., Huang Z.-X., Li X., Shen K.-F., Liu M., Ouyang H.-D., Zhang S.-B., Ruan Y.-T., Zhang X.-L., Wu S.-L. (2018). Upregulation of NLRP3 via STAT3-Dependent Histone Acetylation Contributes to Painful Neuropathy Induced by Bortezomib. Exp. Neurol..

[100] Zhu X.-Y., Huang C.-S., Li Q., Chang R.-M., Song Z.-B., Zou W.-Y., Guo Q.-L. (2012). P300 Exerts an Epigenetic Role in Chronic Neuropathic Pain through Its Acetyltransferase Activity in Rats Following Chronic Constriction Injury (CCI). Mol. Pain.

[101] Li K., Zhao G.-Q., Li L.-Y., Wu G.-Z., Cui S. (2014). Epigenetic Upregulation of Cdk5 in the Dorsal Horn Contributes to Neuropathic Pain in Rats. NeuroReport.

[102] Gu P., Pan Z., Wang X.-M., Sun L., Tai L.W., Cheung C.W. (2018). Histone Deacetylase 5 (HDAC5) Regulates Neuropathic Pain through SRY-Related HMG-Box 10 (SOX10)-Dependent Mechanism in Mice. Pain.

[103] Xie Y., Li Z., Xu H., Ma J., Li T., Shi C., Jin J. (2022). Downregulation of Sp1 Inhibits the Expression of HDAC1/SOX10 to Alleviate Neuropathic Pain-like Behaviors after Spinal Nerve Ligation in Mice. ACS Chem. Neurosci..

[104] Miao J., Chen Z., Wu Y., Hu Q., Ji T. (2022). Sp1 Inhibits PGC-1α via HDAC2-Catalyzed Histone Deacetylation in Chronic Constriction Injury-Induced Neuropathic Pain. ACS Chem. Neurosci..

[105] Zhang J., Chen S.-R., Zhou M.-H., Jin D., Chen H., Wang L., DePinho R.A., Pan H.-L. (2022). HDAC2 in Primary Sensory Neurons Constitutively Restrains Chronic Pain by Repressing A2δ-1 Expression and Associated NMDA Receptor Activity. J. Neurosci..

[106] Liu M., Zhao Y.-T., Lv Y.-Y., Xu T., Li D., Xiong Y.-C., Xin W.-J., Lin S.-Y. (2022). Metformin Relieves Bortezomib-Induced Neuropathic Pain by Regulating AMPKa2-Mediated Autophagy in the Spinal Dorsal Horn. Neurochem. Res..

[107] Liu M., Mai J.-W., Luo D.-X., Liu G.-X., Xu T., Xin W.-J., Lin S.-Y., Li Z.-Y. (2023). NFATc2-Dependent Epigenetic Downregulation of the TSC2/Beclin-1 Pathway Is Involved in Neuropathic Pain Induced by Oxaliplatin. Mol. Pain.

[108] Poulard C., Noureddine L.M., Pruvost L., Le Romancer M. (2021). Structure, Activity, and Function of the Protein Lysine Methyltransferase G9a. Life.

[109] Mo K., Xu H., Gong H., Lei H., Wang Y., Guo W., Xu S., Tu W. (2018). Dorsal Root Ganglia Coactivator-Associated Arginine Methyltransferase 1 Contributes to Peripheral Nerve Injury-Induced Pain Hypersensitivities. Neuroscience.

[110] Liang L., Gu X., Zhao J.-Y., Wu S., Miao X., Xiao J., Mo K., Zhang J., Lutz B.M., Bekker A. (2016). G9a Participates in Nerve Injury-Induced Kcna2 Downregulation in Primary Sensory Neurons. Sci. Rep..

[111] Hsieh M.-C., Ho Y.-C., Lai C.-Y., Wang H.-H., Yang P.-S., Cheng J.-K., Chen G.-D., Ng S.-C., Lee A.-S., Tseng K.-W. (2021). Blocking the Spinal Fbxo3/CARM1/K+ Channel Epigenetic Silencing Pathway as a Strategy for Neuropathic Pain Relief. Neurotherapeutics.

[112] Liang L., Zhao J.-Y., Gu X., Wu S., Mo K., Xiong M., Marie Lutz B., Bekker A., Tao Y.-X. (2016). G9a Inhibits CREB-Triggered Expression of Mu Opioid Receptor in Primary Sensory Neurons Following Peripheral Nerve Injury. Mol. Pain.

[113] Zhang Y., Chen S.-R., Laumet G., Chen H., Pan H.-L. (2016). Nerve Injury Diminishes Opioid Analgesia through Lysine Methyltransferase-Mediated Transcriptional Repression of μ-Opioid Receptors in Primary Sensory Neurons. J. Biol. Chem..

[114] Yadav R., Weng H.-R. (2017). EZH2 Regulates Spinal Neuroinflammation in Rats with Neuropathic Pain. Neuroscience.

[115] An Q., Sun C., Li R., Chen S., Gu X., An S., Wang Z. (2021). Calcitonin Gene-Related Peptide Regulates Spinal Microglial Activation through the Histone H3 Lysine 27 Trimethylation via Enhancer of Zeste Homolog-2 in Rats with Neuropathic Pain. J. Neuroinflammation.

[116] Zhang P., Guergues J., Alleyne A.R., Cirino T.J., Nadeau O., Figueroa A.M., Stacy H.M., Suzuki T., McLaughlin J.P., Stevens S.M. (2022). Novel Histone Modifications in Microglia Derived from a Mouse Model of Chronic Pain. Proteomics.

[117] Rullo L., Franchi S., Amodeo G., Caputi F.F., Verduci B., Losapio L.M., Sacerdote P., Romualdi P., Candeletti S. (2021). Interplay between Prokineticins and Histone Demethylase KDM6A in a Murine Model of Bortezomib-Induced Neuropathy. Int. J. Mol. Sci..

[118] Wang N., Shen X., Bao S., Feng S.-W., Wang W., Liu Y., Wang Y., Wang X., Guo X., Shen R. (2016). Dopaminergic Inhibition by G9a/Glp Complex on Tyrosine Hydroxylase in Nerve Injury-Induced Hypersensitivity. Mol. Pain.

[119] Wang X., Ma S., Wu H., Shen X., Xu S., Guo X., Bolick M.L., Wu S., Wang F. (2018). Macrophage Migration Inhibitory Factor Mediates Peripheral Nerve Injury-Induced Hypersensitivity by Curbing Dopaminergic Descending Inhibition. Exp. Mol. Med..

[120] Tachibana M., Matsumura Y., Fukuda M., Kimura H., Shinkai Y. (2008). G9a/GLP Complexes Independently Mediate H3K9 and DNA Methylation to Silence Transcription. EMBO J..

[121] Li L., Bai L., Yang K., Zhang J., Gao Y., Jiang M., Yang Y., Zhang X., Wang L., Wang X. (2021). KDM6B Epigenetically Regulated-Interleukin-6 Expression in the Dorsal Root Ganglia and Spinal Dorsal Horn Contributes to the Development and Maintenance of Neuropathic Pain Following Peripheral Nerve Injury in Male Rats. Brain. Behav. Immun..

[122] Ghosh K., Zhang G.-F., Chen H., Chen S.-R., Pan H.-L. (2022). Cannabinoid CB2 Receptors Are Upregulated via Bivalent Histone Modifications and Control Primary Afferent Input to the Spinal Cord in Neuropathic Pain. J. Biol. Chem..

[123] Hsieh M.-C., Lai C.-Y., Cho W.-L., Lin L.-T., Yeh C.-M., Yang P.-S., Cheng J.-K., Wang H.-H., Lin K.-H., Nie S.-T. (2023). Phosphate NIMA-Related Kinase 2-Dependent Epigenetic Pathways in Dorsal Root Ganglion Neurons Mediates Paclitaxel-Induced Neuropathic Pain. Anesth. Analg..

[124] Yeh C.-M., Lai C.-Y., Peng H.-Y., Lin T.-B., Chou D., Wang H.-H., Yang P.-S., Cheng J.-K., Peng Y.-C., Hsieh M.-C. (2023). Protein Arginine Methyltransferase 5 Contributes to Paclitaxel-Induced Neuropathic Pain by Activating Transient Receptor Potential Vanilloid 1 Epigenetic Modification in Dorsal Root Ganglion. Anesth. Analg..

[125] Lin T.-B., Lai C.-Y., Hsieh M.-C., Ho Y.-C., Wang H.-H., Yang P.-S., Cheng J.-K., Chen G.-D., Ng S.-C., Peng H.-Y. (2020). Inhibiting MLL1-WDR5 Interaction Ameliorates Neuropathic Allodynia by Attenuating Histone H3 Lysine 4 Trimethylation-Dependent Spinal mGluR5 Transcription. Pain.

[126] Imai S., Ikegami D., Yamashita A., Shimizu T., Narita M., Niikura K., Furuya M., Kobayashi Y., Miyashita K., Okutsu D. (2013). Epigenetic Transcriptional Activation of Monocyte Chemotactic Protein 3 Contributes to Long-Lasting Neuropathic Pain. Brain.

[127] Bell J.T., Loomis A.K., Butcher L.M., Gao F., Zhang B., Hyde C.L., Sun J., Wu H., Ward K., Harris J. (2014). Differential Methylation of the TRPA1 Promoter in Pain Sensitivity. Nat. Commun..

[128] Rajan I., Savelieva K.V., Ye G.-L., Wang C., Malbari M.M., Friddle C., Lanthorn T.H., Zhang W. (2009). Loss of the Putative Catalytic Domain of HDAC4 Leads to Reduced Thermal Nociception and Seizures While Allowing Normal Bone Development. PLoS ONE.

[129] Capasso K.E., Manners M.T., Quershi R.A., Tian Y., Gao R., Hu H., Barrett J.E., Sacan A., Ajit S.K. (2015). Effect of Histone Deacetylase Inhibitor JNJ-26481585 in Pain. J. Mol. Neurosci..

[130] Patnaik E., Madu C., Lu Y. (2023). Epigenetic Modulators as Therapeutic Agents in Cancer. Int. J. Mol. Sci..

[131] Sahafnejad Z., Ramazi S., Allahverdi A. (2023). An Update of Epigenetic Drugs for the Treatment of Cancers and Brain Diseases: A Comprehensive Review. Genes.

